# Pannexin 1 targets mitophagy to mediate renal ischemia/reperfusion injury

**DOI:** 10.1038/s42003-023-05226-x

**Published:** 2023-08-29

**Authors:** Lianjiu Su, Jiahao Zhang, Jing Wang, Xiaozhan Wang, Edward Cao, Chen Yang, Qihao Sun, Ramadoss Sivakumar, Zhiyong Peng

**Affiliations:** 1 Department of Critical Care Medicine, Zhongnan Hospital of Wuhan University, Wuhan, 430071 Hubei China; 2 Clinical Research Center of Hubei Critical Care Medicine, Wuhan, China; 3 Department of Cardiology, David Geffen School of Medicine, University of California, Los Angeles, CA USA; 4 Center of Critical Care Nephrology, Department of Critical Care Medicine, University of Pittsburgh Medical Center, Pittsburgh, PA 15206 USA

**Keywords:** Acute kidney injury, Experimental models of disease, Mechanisms of disease

## Abstract

Renal ischemia/reperfusion (I/R) injury contributes to the development of acute kidney injury (AKI). Kidney is the second organ rich in mitochondrial content next to the heart. Mitochondrial damage substantially contributes for AKI development. Mitophagy eliminates damaged mitochondria from the cells to maintain a healthy mitochondrial population, which plays an important role in AKI. Pannexin 1 (PANX1) channel transmembrane proteins are known to drive inflammation and release of adenosine triphosphate (ATP) during I/R injury. However, the specific role of PANX1 on mitophagy regulation in renal I/R injury remains elusive. In this study, we find that serum level of PANX1 is elevated in patients who developed AKI after cardiac surgery, and the level of PANX1 is positively correlated with serum creatinine and urea nitrogen levels. Using the mouse model of renal I/R injury in vivo and cell-based hypoxia/reoxygenation (H/R) model in vitro, we prove that genetic deletion of PANX1 mitigate the kidney tubular cell death, oxidative stress and mitochondrial damage after I/R injury through enhanced mitophagy. Mechanistically, PANX1 disrupts mitophagy by influencing ATP-P2Y-mTOR signal pathway. These observations provide evidence that PANX1 could be a potential biomarker for AKI and a therapeutic target to alleviate AKI caused by I/R injury.

## Abstract (web summary)

PANX1 which is elevated in serum of AKI patients, mouse I/R injury model and H/R cell models is shown to disrupt mitophagy during I/R injury by influencing the ATP-P2Y-mTOR pathway.

## Introduction

Acute kidney injury (AKI) is characterized by a rapid decline of kidney function, and is usually resulting from renal ischemia/reperfusion (I/R), sepsis, and nephrotoxin. AKI, as a growing public health problem, affects millions of patients each year^1^. When AKI patients progress to dialysis, the mortality rate can reach up to 60–80%^2^. Among survivors, a large percentage of patients will progress to end-stage renal disease or chronic kidney disease. Renal I/R and I/R-AKI still remain severe complications of increased mortality and morbidity of postoperative graft procedures^3^. Considering its complex and unclear pathogenesis^4–6^, further dissecting the molecular mechanism of I/R-AKI is urgently needed to unveil potential drug targets for developing therapeutic strategies.

Accumulating evidence indicates that mitochondrial damage and dysfunction play pivotal roles in the pathogenesis of AKI^7–9^. Not only is cellular metabolism depressed, but damaged or dysfunctional mitochondria also produce excessive reactive oxygen species (ROS) and release apoptosis factors^10^. Emerging evidence indicated that mitophagy is implicated in AKI, which can arise from nephrotoxins, sepsis, ischemia/reperfusion, and chronic kidney diseases^11^. Mitophagy is induced as an adaptive or defense mechanism for maintaining a population of healthy mitochondria and thereby ensuring cell survival^11,12^. So far, the PINK1-PARKIN pathway is recognized as the most well-described classic pathway in mitophagy^11–15^. Therefore, protection and maintenance of mitochondrial function have been proposed as measures to treat renal I/R injury or AKI.

Multiple studies have reported the contribution of PANX1 channels in inflammation and injury responses^16^. PANX1 is confirmed to be the regulator of ATP release as a damage-associated molecular pattern^17,18^. Importantly, our previous research demonstrated that PANX1 deletion protects against I/R-AKI by limiting ferroptosis-mediated oxidative damage^19^. In oxidative environments, PANX1 activates apoptosis or autophagy signaling by increasing the efflux of extracellular ATP, which can activate the purinergic P2X and P2Y signaling pathway^20^. Furthermore, P2Y receptors can promote mechanistic target of rapamycin (mTOR) signaling which augments mitochondrial activity^21^. Thus, mTOR signaling can directly regulate mitochondrial function and mitophagy^22,23^. In the present study, we hypothesize that PANX1 regulates the P2Y receptor by releasing ATP, thereby regulating the mTOR pathway and affecting mitophagy in renal I/R injury.

Considering the regulatory effects of PANX1 on cellular events, which directly correlate with the progression of kidney I/R injury, we examined the PANX1 knockout mice to evaluate the potential role and underlying mechanisms of PANX1 mediated renal I/R injury, specifically on mitophagy and related cell signaling.

## Results

### Serum PANX1 is elevated in AKI patients after cardiac surgery

**Fig. 1:**
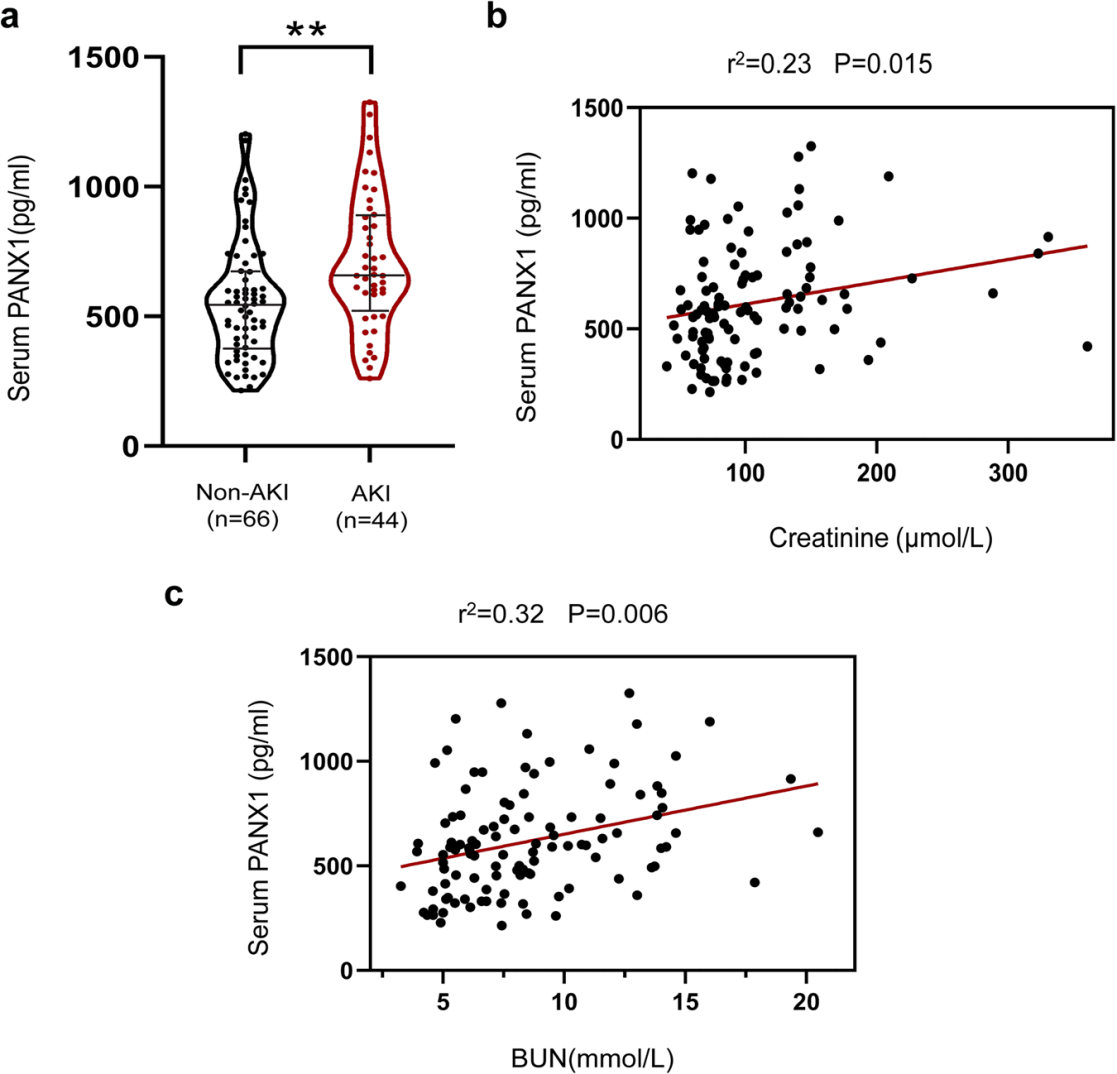
Serum PANX1 level of heart surgery patients. **a** The level of PANX1 in the serum of patients experiencing AKI or non-AKI after heart surgery. Results are presented as means ± SEM. ** indicates *p* < 0.01 compared with the non-AKI patients from Mann–Whitney *U* tests. **b**, **c** The correlation between serum PANX1 levels and serum creatinine and blood urea nitrogen in patients with AKI after cardiac surgery. *P* values from Pearson’s Correlation.

To explore whether PANX1 is involved in I/R-induced AKI, we evaluated 110 patients who had undergone cardiac surgery. They were separated into two groups based on the presence (AKI) or absence of AKI (non-AKI) as determined by serum creatinine and urea. As shown in Fig. 1a, serum PANX1 level was elevated in AKI patients than that of non-AKI patients. Furthermore, we found that there was a clear positive correlation between serum PANX1 levels and serum creatinine (*P* = 0.015) and urea nitrogen (*P* = 0.006, Fig. 1b, c). These results suggested that increased expression of PANX1 was associated with AKI after cardiac surgery and PANX1 could be a biomarker for AKI, in addition to serum creatinine and urea nitrogen.

### PANX1 expression levels are increased during kidney I/R injury in vivo and in vitro

**Fig. 2:**
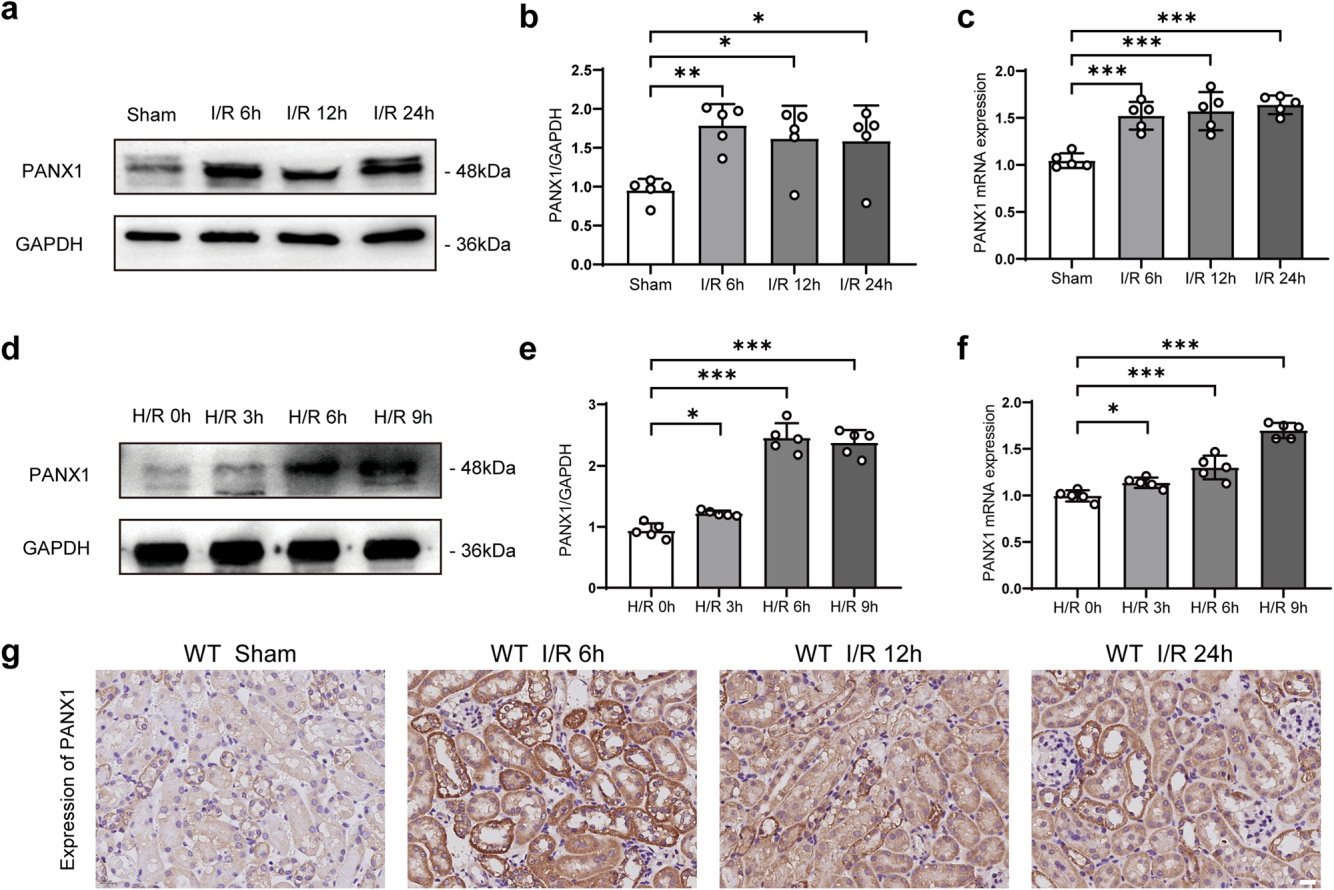
PANX1 expression associated with I/R-induced kidney injury. **a**, **b** Western blot measuring the PANX1 protein in kidney of mice subjected to sham treatment or ischemia/reperfusion for the indicated times (*n* = 5). **c** PANX1 mRNA expression in kidney of mice subjected to sham treatment or ischemia/reperfusion for the indicated times (*n* = 5). **d**, **e** PANX1 protein expression in HK2 cells after control or hypoxia/reoxygenation for indicated time points (*n* = 5). **f** PANX1 mRNA expression in HK2 cells after control or hypoxia/reoxygenation for indicated time points (*n* = 5). **g** Representative immunohistochemical staining images of renal tissue show the expression of PANX1 protein (Bar = 50 μm). Results are presented as means ± SEM. *P* values from One-way ANOVA with Dunnett’s multiple comparisons test. * indicates *p* < 0.05; ** indicates *p* < 0.01; *** indicates *p* < 0.001.

To investigate the relationship between PANX1 and kidney I/R injury in vivo, a renal I/R mouse model was established by subjecting mice to a renal artery blockage followed by reperfusion after a specified time period (6 h, 12 h, or 24 h). Mice that were subjected to the same procedure but without an arterial blockage served as sham controls. As shown in Fig. 2a, b, PANX1 protein expression was strongly induced at 6 h after I/R injury and remained elevated up to 24 h compared to the sham-operated group, which was consistent with our previous research^19^. We also determined the transcriptional level of PANX1, which increased after I/R injury (Fig. 2c). Additionally, we challenged the human kidney-2 (HK2) cells by exposing them to hypoxia/reoxygenation (H/R) injury for different lengths of time (reoxygenation 60 minutes after hypoxia 0 h, 3 h, 6 h, or 9 h). Concurrent with the in vivo data, PANX1 expression was strongly induced by H/R injury in a time-dependent manner (Fig. 2d, e). We also determined the transcriptional level of PANX1, which increased after hypoxia (Fig. 2f). In line with these observations, immunohistochemical staining of renal tissue with anti-PANX1 antibody clearly showed that PANX1 protein was increased after I/R injury as compared with the sham control group (Fig. 2g). We also performed laser confocal detection of PANX1, and plasma membrane or endoplasmic reticulum staining. The results also showed an increase of PANX1 (Supplementary Fig. 1a, b). Taken together, these results indicate that PANX1 expression is increased following I/R injury and might play an important role in I/R injury and AKI development.

### PANX1 deletion decreased kidney tubular cell death and alleviated kidney injury

**Fig. 3:**
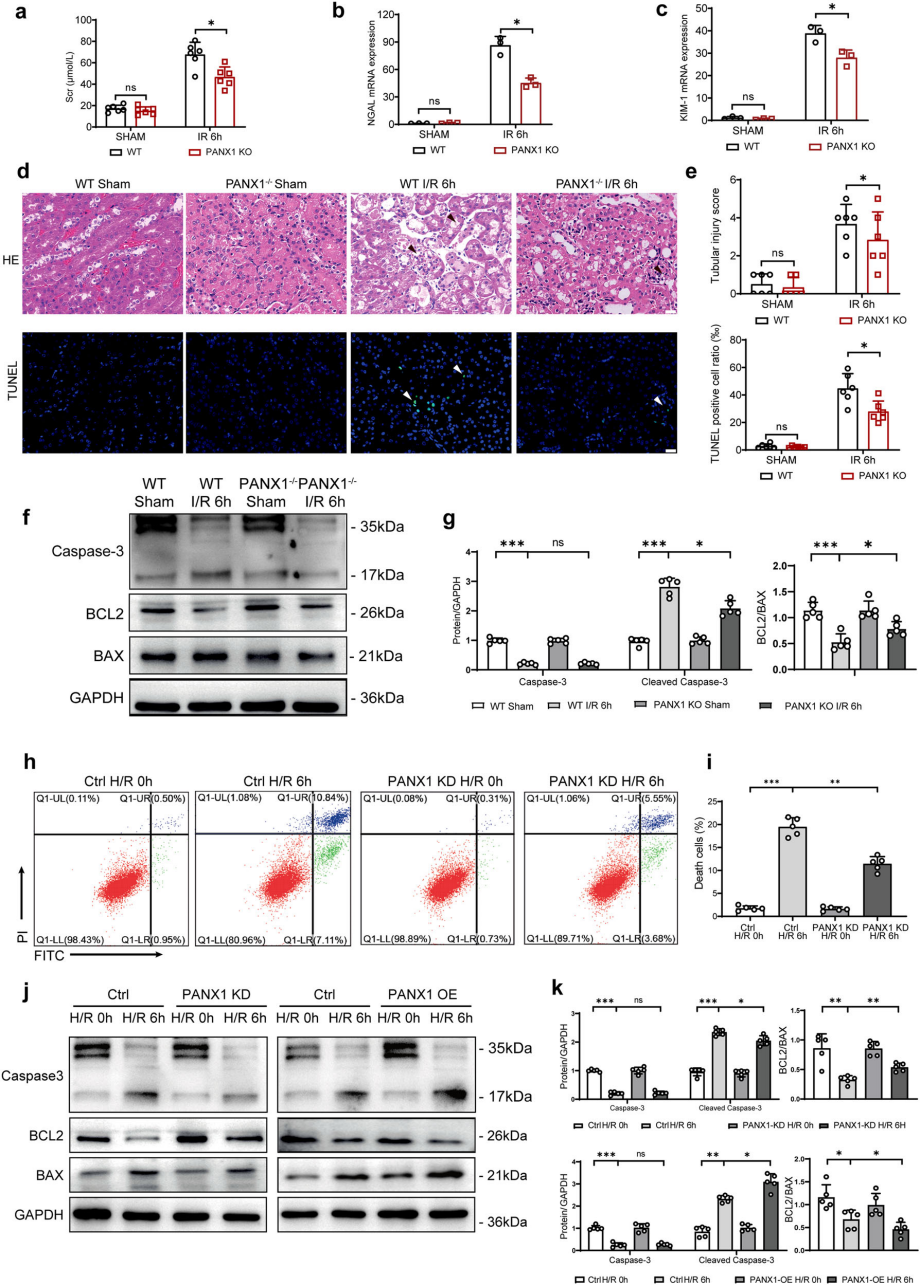
PANX1 knockout alleviates mice kidney I/R injury. **a** Serum creatinine (Scr) levels of PANX1−/− mice and WT mice subjected to sham or I/R treatment (*n* = 6). **b** NGAL mRNA levels of PANX1−/− mice and WT mice subjected to sham or I/R treatment (*n* = 3). **c** KIM-1 mRNA levels of PANX1−/− mice and WT mice subjected to sham or I/R treatment (*n* = 3). **d** Representative histological H&E staining and TUNEL staining show necrotic areas in kidneys from PANX1−/− mice and corresponding WT mice subjected to sham or I/R operation (Bar = 50 μm). **e** Tubular injury scores and TUNEL-positive cells rate (*n* = 6). **f**, **g** The protein levels of apoptosis-related protein in PANX1−/− and WT mice subjected to sham or I/R challenge (*n* = 5). **h**, **i** Representative histogram showing apoptosis for HK2 and PANX1KD cells from the indicated groups measured by flow cytometry (*n* = 5). **j**, **k** The protein levels of apoptosis-related protein in PANX1KD and PANX1OE cells subjected to normal or H/R challenge (*n* = 5). GAPDH served as the loading control. Results are presented as means ± SEM. *P* values from Student’s *t* test. * indicates *p* < 0.05; ** indicates *p* < 0.01; *** indicates *p* < 0.001.

Our clinical data, mouse model of I/R injury and cell-based H/R assay all consistently showed the increased PANX1 expression, as described in our previous study, we have studied the regulatory effect of PANX1 on AKI through ferroptosis at a later time point (24 h)^19^. Considering the different effects of autophagy and ferroptosis at different stages of AKI^24^, we detected changes in kidney at an earlier time point (6 h). We generated mice that are globally deficient in PANX1 (PANX1−/−) (Supplementary Fig. 1c) and subjected them to I/R kidney injury. As presented in Fig. 3a, serum creatinine (Scr) was increased in I/R groups compared with sham groups, which is indicative of successful kidney injury. Interestingly, Scr levels in PANX1 knockout (KO) mice were lower than the wild-type (WT) mice after I/R. This indicates that a PANX1 could be a key player that control I/R injury in kidney. Additionally, we tested the levels of NGAL and KIM-1, which represent the early biomarkers of AKI and found that after PANX1 KO, the levels of NGAL and KIM-1 were both decreased compared with WT mice after I/R (Fig. 3b, c). Altogether, these results showed that PANX1 KO potentially decreased the levels of AKI biomarkers following I/R injury. Next, we asked whether PANX1 deficiency protect the glomerular injury and apoptosis after I/R. As expected, glomerular injury and apoptosis were higher in I/R groups than sham groups. Notably, glomerular injury and apoptosis in PANX1−/− mice were observed to be reduced than in WT mice after injury (Fig. 3d, e). Next, we examined the classical apoptotic and anti-apoptotic markers expression after I/R injury. We found that the expression of apoptotic markers such as BAX and Cleaved Caspase 3 were increased and anti-apoptotic protein BCL-2 was decreased after I/R injury in both WT and PANX1−/− mice. However, PANX1 deletion reduced the effects of I/R injury induced changes in apoptotic and anti-apoptotic protein expressions (Fig. 3f, g). These observations suggest that PANX1 might be a mediator of I/R injury induced renal apoptosis.

Next to examine, whether in vitro H/R model could recapitulate the effects PANX1 deletion on in vivo I/R injury, we utilized both loss and gain of function experiments by depleting and overexpressing PANX1 in HK2 cells, respectively (Supplementary Fig. 1d–g) and subjecting them to H/R injury. First, we checked that apoptotic cell death in HK2 cells depleted for PANX1 by flow cytometry. As expected, H/R injury increased apoptotic cell death in both scrambled control and PANX1-depleted cells. However, the magnitude of apoptosis induction was diminished in HK2 depleted for PANX1 (Fig. 3h, i). Consistently, H/R-induced changes in the expression of pro-apoptotic proteins BAX and Cleaved Caspase 3 and anti-apoptotic BCL-2 were mitigated by PANX1 knockdown (Fig. 3j, k). Conversely, PANX1 overexpression exacerbated the effects of H/R injury-induced changes in HK2 cells as the induction of BAX and Cleaved Caspase 3 proteins and reduction in BCL-2 expression were pronounced much in HK2 cells overexpressing PANX1 compared to empty vector control (Fig. 3j, k). These results suggest that PANX1 mediates the H/R-induced apoptosis cell death in HK2 cells.

### PANX1 deficiency alleviates oxidative stress by reducing the mitochondrial damage

**Fig. 4:**
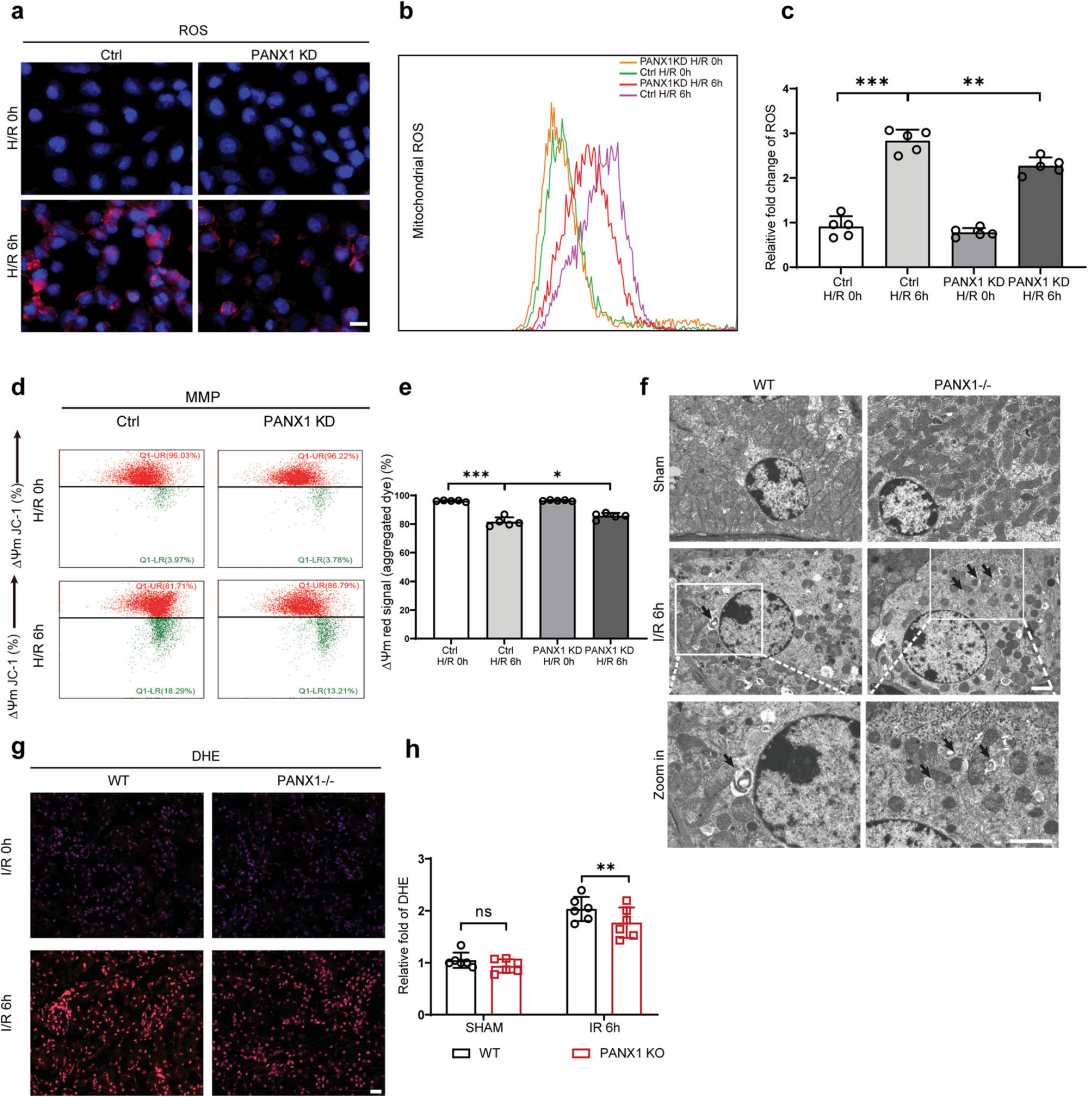
PANX1 deficiency alleviates oxidative stress and mitochondrial damage in hypoxia/reoxygenation HK2 cells. **a** Immunofluorescence of ROS from PANX1KD and HK2 cells after H/R injury (Bar = 20 μm). **b**, **c** Representative histogram showing mitochondrial ROS for HK2 and PANX1KD cells from the indicated groups measured by flow cytometry (*n* = 5). **d**, **e** Representative mitochondrial membrane potential (MMP) in H/R or normal HK2 cells from the indicated groups measured by flow cytometry (*n* = 5). **f** Representative images of transmission electron microscopy in different groups, arrows represent mitochondria undergoing mitophagy (Bar = 5 μm). **g**, **h** DHE staining for ROS in different groups (Bar = 20 μm) (*n* = 5). Results are presented as means ± SEM. *P* values from Student’s *t* test. * indicates *p* < 0.05; ** indicates *p* < 0.01; *** indicates *p* < 0.001.

Previous studies have shown that organ I/R and cell H/R are accompanied by oxidative stress and release of reactive oxygen species, which promotes apoptotic and necrotic cell deaths. Therefore, we hypothesized that PANX1 may mediates its effect through mitochondrial damage during I/R and H/R injury. To test our hypothesis, we analyzed mitochondrial damage and oxidative stress in HK2 cells exposed to H/R under normal or PANX1-depleted condition. After H/R injury, mitochondrial ROS production increased in HK2 cells compared to control cells. However, PANX1 depletion suppressed the H/R-induced ROS production in HK2 cells (Fig. 4a). Concurrent with this observation, mitochondrial ROS production was also diminished by PANX1 depletion following H/R injury as determined by flow cytometry (Fig. 4b, c). Overall, we conclude that PANX1 knockdown can alleviate mitochondria ROS generation in HK2 cells in response to H/R injury.

Mitochondria plays an important role in cellular oxygen and energy metabolism. Normal mitochondrial membrane potential (MMP) is crucial to maintaining normal physiological functions of cells^25^. Correspondingly, the level of MMP can reflect mitochondria injury. In our study, the level of MMP in HK2 cells decreased during H/R injury, representing the induction of mitochondrial membrane damage. Interestingly, PANX1 deficiency reduced the decrease in MMP induced by H/R injury and thus indicating the reduced mitochondrial membrane damage under PANX1 depleted condition (Fig. 4d, e).

Mitochondrial number and condition are the crucial factor that determine the normal homeostasis of the cells. We utilized transmission electron microscope to check the mitochondrial dynamics in kidney. We observed the high abundance and healthy mitochondria in sham groups but I/R injury not only reduced the mitochondrial number but caused mitochondrial swelling, deformation, matrix thinning and decreased cristae. Surprisingly, PANX1 depletion further enhanced mitophagy and prevented mitochondrial damage (Fig. 4f). In addition, we also performed DHE staining to detect ROS in kidney after I/R injury. Indeed, we found renal oxidative stress after I/R injury in both WT and PANX1−/− mice. Intriguingly, PANX1−/− mice had reduced superoxide levels than WT mice after I/R injury (Fig. 4g, h).

### PANX1 regulates mitophagy during renal ischemia-reperfusion injury process

**Fig. 5:**
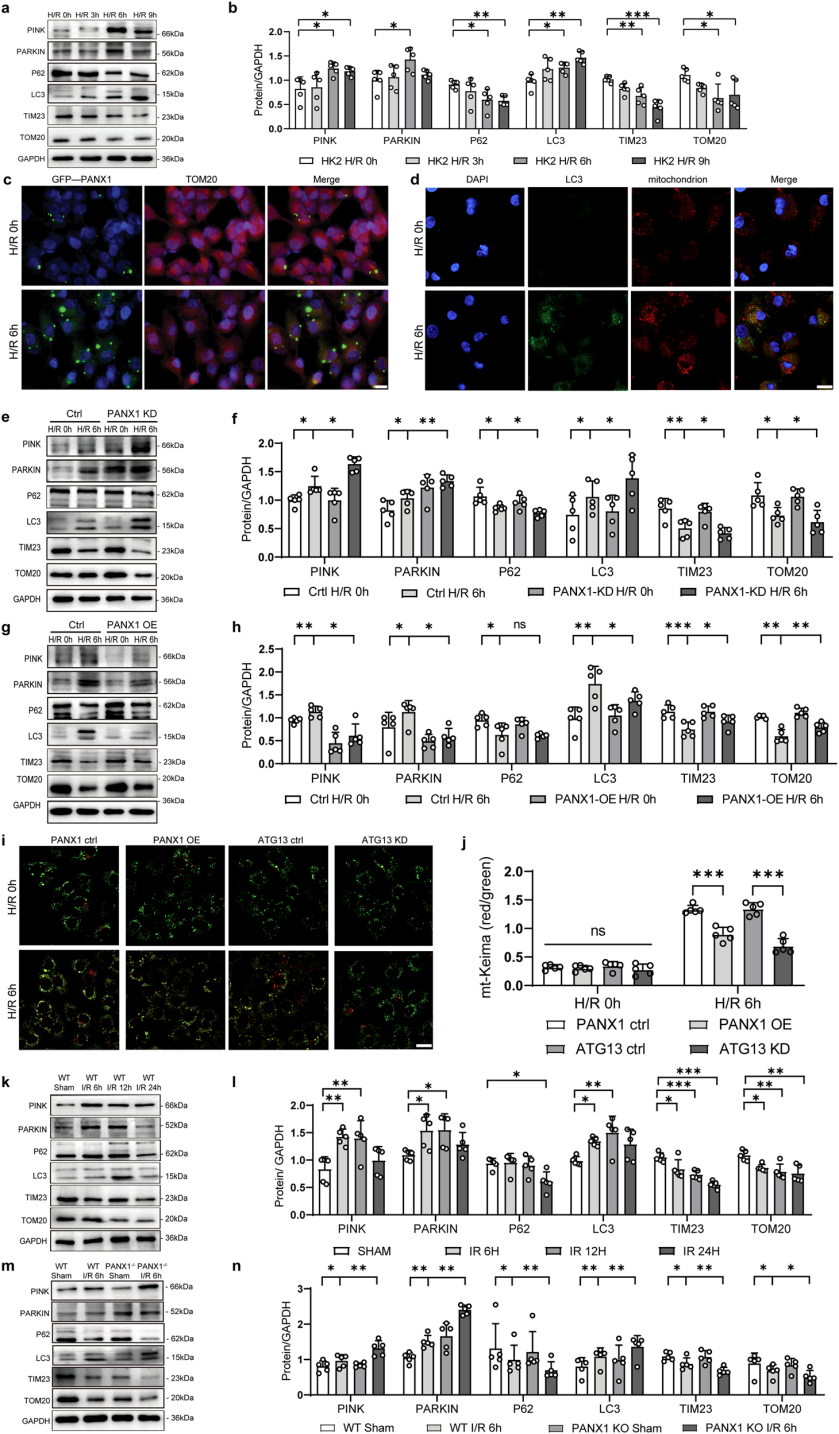
PANX1 regulates mitophagy during renal ischemia reperfusion injury process. **a**, **b** Western blot measured the protein levels of mitophagy-related proteins in HK2 cells subjected to H/R for the indicated time points (*n* = 5). Results are presented as means ± SEM. *P* values from One-way ANOVA with Dunnett’s multiple comparisons test. **c** Representative images of fluorescence of PANX1 and TOM20 protein from H/R or control (Bar = 20 μm). **d** Laser confocal scanning microscopy for LC3 and mitochondrion staining (Bar = 20 μm). **e**–**h** Western blot indicated the levels of mitophagy-related proteins in PANX1KD and PANX1OE cells treated with H/R or normal (*n* = 5). Results are presented as means ± SEM. *P* values from Student’s *t* test. **i**, **j** Mitochondrial Keima (mt-Keima) analysis of mitophagy response in the process of hypoxia and reoxygenation (Bar = 20 μm) (*n* = 5). Results are presented as means ± SEM. *P* values from Student’s *t* test. **k**, **l** Western blot analysis of mitophagy-related proteins expression in mice subjected to renal I/R operation for the indicated time points (*n* = 5). Results are presented as means ± SEM. *P* values from one-way ANOVA with Dunnett’s multiple comparisons test. **m**, **n** The levels of mitophagy-related protein in the kidney of the indicated PANX1 knockdown mouse groups after I/R insult (*n* = 5). Results are presented as means ± SEM. *P* values from Student’s *t* test. GAPDH served as the loading control. * indicates *p* < 0.05; ** indicates *p* < 0.01; *** indicates *p* < 0.001.

Under stressful conditions, ATP has been reported to engage in the generation of mitochondrial ROS, the release of mitochondrial DNA and the decrease of MMP. The decrease of MMP promotes the accumulation of PINK on the outer mitochondrial membrane. PINK1 will phosphorylate PRKN on the Ub-like domain on the Ser resulting in an increase of its E3 activity^10^. Activated PRKN polyubiquitinates numerous substrates of OMM proteins, leading to the recruitment of the autophagy machinery^12^. Considering the role of PANX1 in the regulation of ATP release, and given our finding that PANX1 regulates mitochondrial number and structure under H/R conditions, we assume that PANX1 may be involved in the regulation of mitophagy. To check this notion, we measured the expressions of various mitophagy-related proteins in the H/R exposed HK2 cells for different reperfusion time periods. The expressions of TOM20 and TIM23 protein located on mitochondria were reduced and while the levels of PINK and PARKIN, which regulate mitophagy, were increased during H/R injury (Fig. 5a, b). Meanwhile, autophagic protein LC3 was increased during H/R injury in all time points studied. On the other hand, autophagic carrier protein P62 was decreased to form autophagosomes (Fig. 5a, b). TOM20 is expressed on the outer membrane of mitochondria and plays an important role in the process of mitophagy. In our study, we found that after H/R injury, the expression of PANX1 increased and the expression of TOM20 decreased in HK2 cells compared with controls (Fig. 5c). The decrease in TOM20 after H/R injury represented a decrease in mitochondria caused by mitophagy. Additionally, we performed confocal analysis for LC3 and mitochondria, and found that H/R injury can increase the accumulation of LC3 in mitochondria (Fig. 5d).

To explore the relationship between PANX1 and mitophagy-related proteins, we performed western blot in PANX1 knockdown and overexpressed HK2 cells that are subjected to H/R injury (Fig. 5e–h). H/R stress-induced changes in the expression of mitophagy-related proteins (PINK, PARKIN and LC3 increased while P62, TIM23, and TOM20 decreased) were enhanced by PANX1 knockdown in HK2 cells at 6 h time point compared with scrambled control HK2 cells (Fig. 5e, f). In contrast, PANX1 overexpression further inhibited the H/R injury induced changes in mitophagy-related protein expressions in HK2 cells (Fig. 5g, h). We also used CCCP to determine whether PANX1 inhibits mitophagy induced by mitochondrial stress. After 1 hour of 100 µM CCCP treatment, there was a change in PANX1(Supplementary Fig. 2a, b). At the 3 hours interval, knockdown of PANX1 demonstrated enhanced CCCP induced mitophagy in HK2 cells. Conversely, PANX1 overexpression resulted in decreased CCCP-induced mitophagy in HK2 cells (Supplementary Fig. 2c–f). These findings suggest that PANX1 is the negative regulator of mitophagy and thus prevents the clearance of damaged mitochondria from the cells.

Changes in mitophagy-related proteins prompted us to further explore whether there were corresponding changes in mitophagy. Mitochondrial Keima (mt-Keima) was used in this study to track mitochondrial trafficking into the lysosome through the mitophagy pathway, which can intuitively reflect the degree of mitophagy. We observed a heightened mitophagy response following H/R in HK2 cells transfected with empty vector. However, overexpression of PANX1 in HK2 cells reduced the mitophagy response, which is similar to findings in ATG13 knockdowns (Fig. 5i, j and Supplementary Fig. 1h). Similarly, I/R injury in PANX1−/− mice showed higher mitophagy response compared to WT mice (Fig. 5k–n).

### PANX1 regulates mitophagy through ATP-P2Y-mTOR pathway

**Fig. 6:**
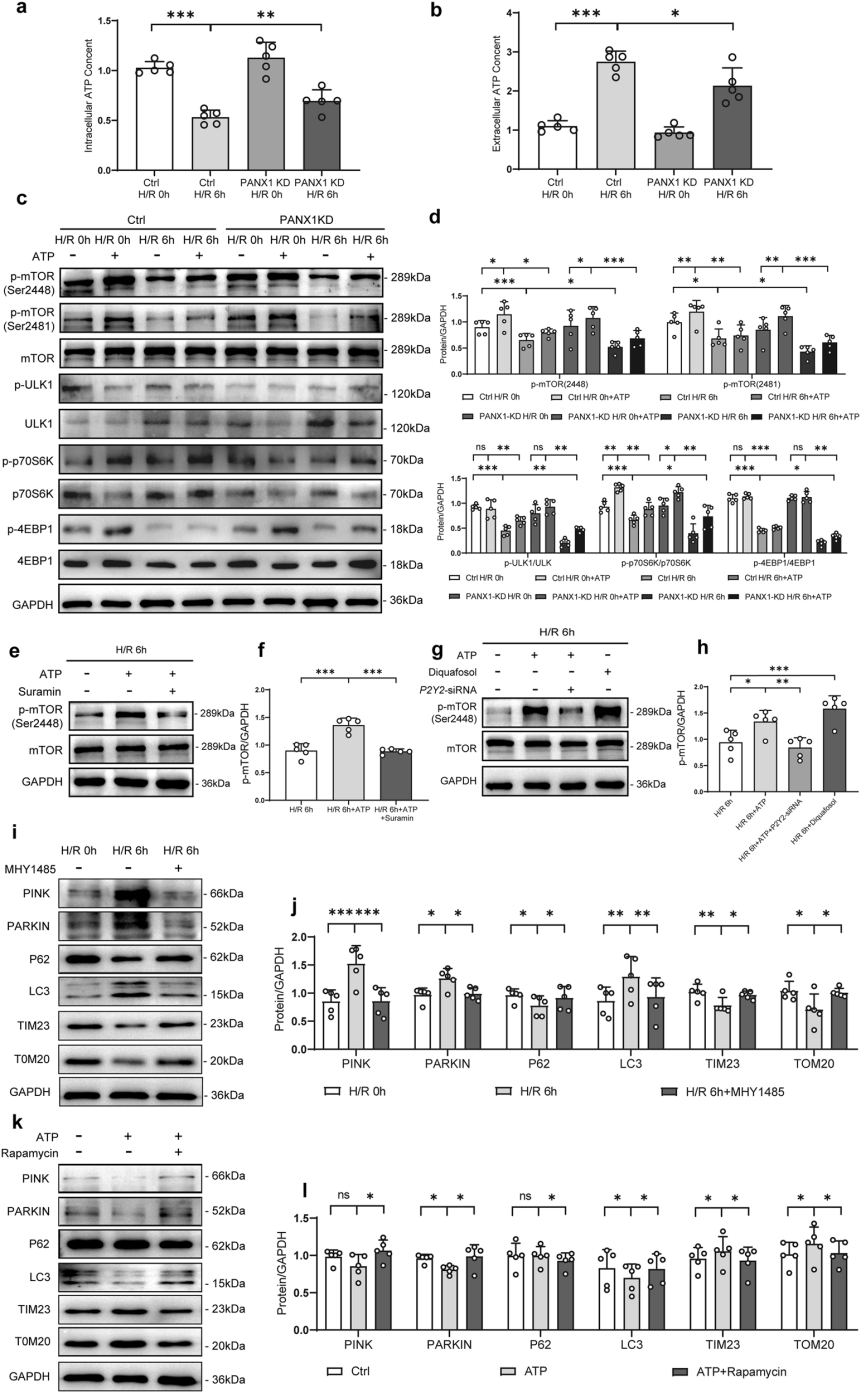
PANX1 regulates mitophagy through ATP-P2Y-mTOR pathway. **a**, **b** Intracellular and extracellular ATP content of PANX1KD and HK2 cells in H/R 6 h and control (*n* = 5). **c**, **d** The expression of Phosphorylation of mTOR substrates in HK2 cells of PANX1KD or control treated with ATP or not under H/R condition (*n* = 5). **e**–**h** Protein levels of mTOR obtained from HK2 H/R cells treated with inhibitors (Suramin) or agonists (Diquafosol tetrasodium) of the P2Y2 receptor (*n* = 5). **i**–**l** The expression of mitophagy-related proteins in the mTOR pathway inhibitor (rapamycin) and activator (MHY1485) treated HK2 cells were measured in the indicated groups (*n* = 5). GAPDH served as the loading control. Results are presented as means ± SEM. *P* values from Student’s *t* test. * indicates *p* < 0.05; ** indicates *p* < 0.01; *** indicates *p* < 0.001.

ATP, as chemical messengers, is cross-linked with other transmitter networks to coordinate numerous aspects of cell behaviors such as proliferation, differentiation, migration, apoptosis, and other physiological processes critical for the proper function of organisms^26^. In our study, we found that intracellular ATP contents were decreased and extracellular ATP contents were increased in H/R injury compared with normal controls (Fig. 6a, b). Intriguingly, intracellular ATP contents in PANX1 knockdown HK2 cells were much higher than that of control HK2 cells (Fig. 6a), and the extracellular ATP contents in PANX1 knockdown HK2 cells were much lower than that of control HK2 cells (Fig. 6b). This indicates that PANX1 regulates ATP release in response to H/R stress possibly by inhibiting ATP channels.

Considering the important role of PANX1 in the regulation of ATP release, we wanted to check whether PANX1 is involved in the regulation of ATP-sensitive cell signaling pathway during I/R-AKI. Since mTOR signaling is known to augment mitochondrial activity after P2Y receptor activation^21,27^, we examined the effect of ATP addition on mTOR expression and phosphorylation of mTOR substrates under normal and H/R conditions in HK2 cells. We observed that ATP could activate mTOR signaling and this effect could be inhibited by H/R injury, indicating that H/R injury could directly inhibit mTOR signaling. Furthermore, PANX1 knockdown in HK2 cells decrease the of mTOR signaling activated by H/R injury by regulating the level of extracellular ATP content (Fig. 6c, d).

Since p-mTOR 2448 and p-mTOR 2481 can be activated by P2Y receptors, as reported by earlier findings^21,27^, we further investigated whether ATP activated mTOR through the P2Y pathway. As shown in Fig. 6e, f, ATP could induce the phosphorylation of mTOR at serine 2448 in response to H/R injury and concomitant incubation with P2Y receptor blocker (Suramin) abrogated the same in HK2 cells. Additionally, siRNA mediated knockdown of P2Y2 also inhibited ATP-induced mTOR phosphorylation (Fig. 6g, h and Supplementary Fig. 1i). Conversely, a P2Y2 activator (Diquefosol) increased the expression of p-mTOR under basal and ATP stimulated conditions in HK2 cells subjected to H/R injury (Fig. 6g, h). Altogether, these observations show that the ATP can activate mTOR phosphorylation in H/R injury through P2Y2 signaling pathway.

Notably, as ATP content regulated the mTOR pathway, mitophagy-related proteins showed corresponding changes similar to that observed with mTOR (Fig. 6i–l). To further verify the effect of mTOR on mitophagy, we used rapamycin to inhibit the mTOR pathway and MHY1485 to enhance the mTOR pathway. We found that H/R injury could activate mitophagy while the effect of activation could be inhibited by mTOR activator MHY1485 (Fig. 6i, j). On the contrary, while ATP could inhibit the mitophagy after H/R injury, this inhibition was reduced when p-mTOR inhibitor Rapamycin was added to the HK2 cells (Fig. 6k, l). This demonstrated that activation of p-mTOR could inhibit mitophagy in H/R injury.

## Discussion

The PANX1 channel plays a key role in oxidative stress, inflammation, and cell death via mechanical stimulation and/or posttranslational modifications^28^. In this study, we provided evidence that PANX1 plays an important role in renal I/R injury by inhibiting mitophagy, inducing oxidative stress and cell death. Therefore, our study has important implication in targeting PANX1 in AKI to alleviate the renal damage and to promote the tubular repair. Additionally, we also reported that PANX1 was elevated in the serum of patients who developed AKI after cardiac surgery. This finding indicates that PANX1 could be a candidate biomarker to diagnose AKI in patients and the severity of AKI could be also be predicted by the level of PANX1 in the serum. Although PANX serves as a membrane channel protein, a relevant study has proven that cleavage of the C terminus is required for PANX1 activation^29^. The cleaved peptide segments may be released into the serum for detection, and the serum PANX1 concentration is proportional to the activated PANX protein. Relevant studies have shown that serum PANX1 concentration has predictive value for the prognosis of acute supratentorial intracerebral hemorrhage and traumatic brain injury^30,31^. Mitophagy served as critical component of mitochondrial quality control by removing damaged/dysfunctional mitochondria from the cell to ensure normal cellular homeostasis and to support cell viability. Accumulating evidences links the impaired mitophagy with disease pathogenesis/progression in various pathological conditions including kidney diseases^10^. Renal tissue possesses enormous mitochondrial content next to heart and thus mitochondrial biogenesis and mitophagy are critical for renal tissues to overcome the stressful conditions including I/R injury and AKI. In this regard, our findings uncovered a mechanism of mitophagy regulation in renal tissue by the PANX1 channel protein. Moreover, we provided substantial evidence that PANX1 played a central role in mediating I/R injury induced renal damage and apoptosis by targeting two important mitochondrial dependent mechanisms such as ROS production and mitophagy.

The PANX1 channel is one of the major ATP release pathways, and extracellular ATP binds to P2Y receptor to further regulate apoptosis and autophagy-related signaling^17,18^. A previous study has reported that in the process of AKI, PANX1 channel-mediated ATP release not only promotes inflammatory injury but also inhibits tissue repair^20^. As a paracrine molecule, extracellular ATP can activate P2Y receptors, which can regulate various signaling pathways^17,18,32^. In addition, P2Y2 can promote the activation of mTOR and mitochondria, and P2Y2 can even directly induce mitochondrial damage^21,33^. Several studies have shown that mTOR signaling could directly regulate mitochondrial function^22,23^. For instance, blocking mTOR signaling with rapamycin inhibitor, PP242 or blocking mitochondrial ATP production (e.g., with CCCP) reduced mitochondrial Ca2+ uptake and impaired cellular ATP release and neutrophil chemotaxis^21^. Indeed, our mechanistic studies indicated that PANX1 indirectly regulates P2Y-mTOR signaling through ATP release from the cells to regulate mitophagy.

Many studies have shown that mTOR signaling pathway is involved in regulating mitophagy or autophagy^34–36^. Yang, X., et al. found that resveratrol promoted SIRT1 (Sirtuin1)-induced mitophagy activation target of mTOR pathway^37^. Mitophagy is inhibited by the mTOR pathway through activation of mTOR complex 1 (mTORC1)^38^. In brown adipose tissue, decreased mTOR activity can stimulate mitophagy^38^. Therefore, down-regulation of mTORC1 is necessary, if not sufficient, for induction of mitophagy^39^.

The effect of mTORC1 on the PINK1-PARKIN signaling in regulating mitophagy was confirmed. The activation of the PINK1-PARKIN pathway recruited many proteins on mitochondria^40^, which was activated and further recruited downstream ATG proteins to coordinate mitophagy^41^. Moreover, mTORC1 negatively regulates autophagy in multiple ways^42–44^. Bordi, M established that mTOR inhibition both reversed autophagy and restored mitophagy level^45^. In the present study, we found that PANX mainly acts as an ATP channel to regulate mitochondrial autophagy by measuring the concentration of intracellular and extracellular ATP and by culturing cells with exogenous ATP. ATP has been reported to engage in various signaling cascades^46^, directly damaging mitochondria through P2Y receptors, leading to the generation of mitochondrial ROS and the release of mitochondrial DNA^33^. Although mTOR has regulatory effects on both mitophagy and autophagy, in the present study, we mainly observed that PANX1 deficiency can enhance mitophagy by regulating ATP outflow.

However, it remains unclear whether ATP release is the only way for PANX1 to regulate mTOR signaling to regulate mitophagy. Earlier studies indicate that PANX1 is present in the endoplasmic reticulum membrane, where it is believed to act as a leaky Ca2+ channel^47,48^. Furthermore, mitochondria play a crucial role in regulating intracellular calcium transport and ion levels by providing ATP for calcium transporting proteins and calcium signaling^49,50^. Whether PANX1 can regulate mitochondrial autophagy by regulating calcium ion levels and downstream mTOR pathway needs to be further investigated. On the other hand, after ATP efflux, the reduced intracellular ATP pool can lead to energy crisis and metabolic stress, damaging and reducing basic cell functions in the kidneys^20^. Metabolic stress results in mitochondrial dysfunction, ROS production increasing, apoptosis, and necrosis, all of which are key determinants of AKI severity.

Our research had some limitations. Firstly, PANX1 was not only distributed in the cell membrane, but also expressed on the surface of membrane organelles such as the endoplasmic reticulum and mitochondrial membrane. Although the expression of PANX1 on the membrane surface of organelles in cells was low, our study did not exclude the influence of PANX1 on the membrane surface of organelles, and only studied the role of PAXN1 on the membrane surface in I/R-induced AKI. Secondly, we used PANX1 whole-organism knockout mice, but did not use kidney specific PANX1 knockout mice. Due to the crosstalk interaction between organs, we could not fully understand the role of PANX1 in the biological process of I/R-induced AKI. Finally, we simply observed that PANX1 in the serum samples of patients after cardiopulmonary bypass surgery can be increased in the early postoperative period. We did not conduct a multifactorial analysis to exclude confounding factors, and we could not determine that PANX1 is an independent risk factor for I/R-induced AKI or just a concomitant increase.

**Fig. 7:**
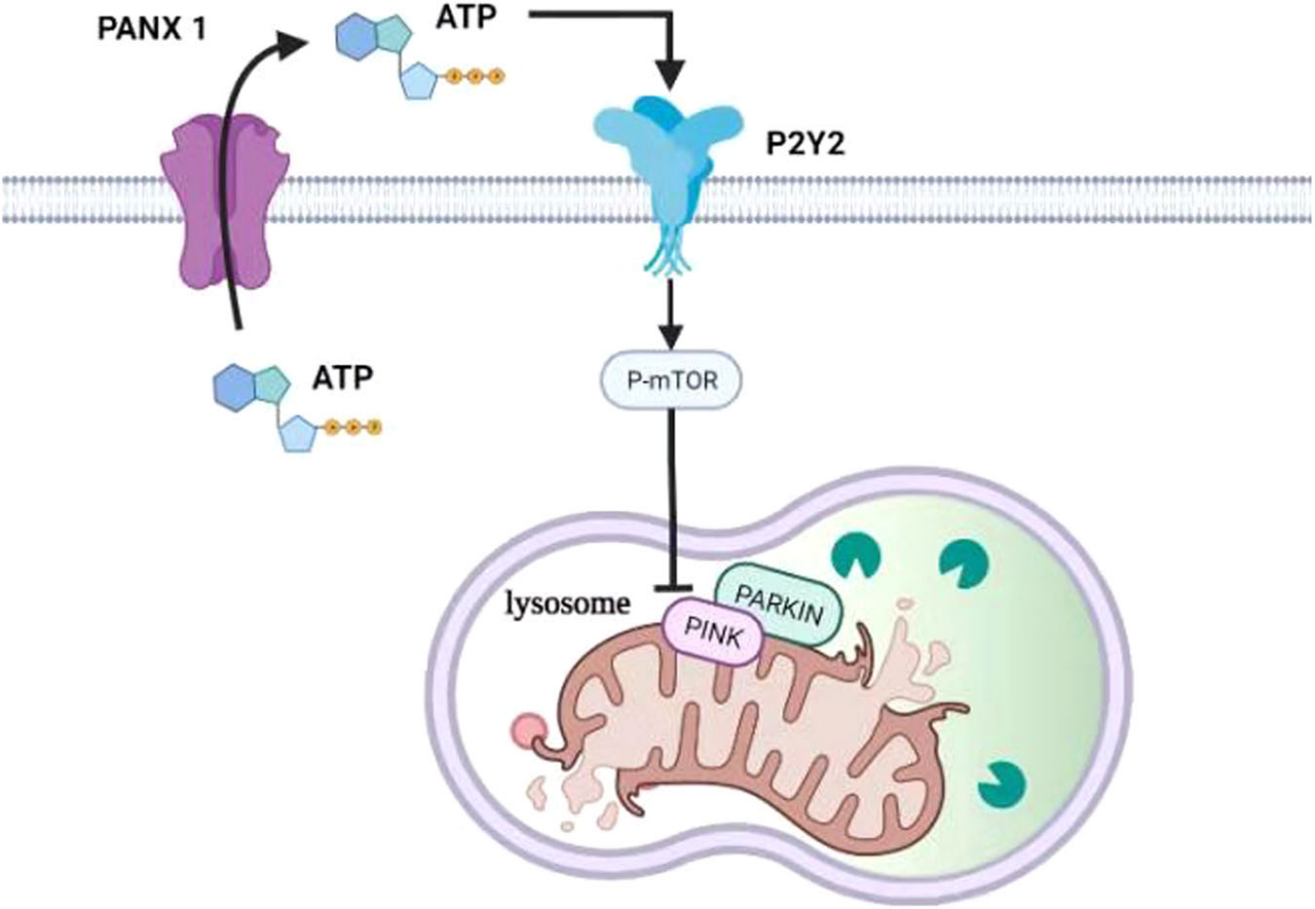
The signal pathway of PANX1 in regulating mitophagy in AKI. PANX1 releases ATP which binds to receptor of P2Y2. The P2Y2 activates mTOR signaling pathway which inhibits the Parking-dependent mitophagy.

In summary, we showed the negative role of PANX1 channel on kidney mitophagy in response to I/R injury. PANX1 channel promotes ATP release to activate P2Y receptors and thereby activate mTOR signaling, which ultimately inhibited mitophagy. PANX1 channels could evidently serve as a central player in the development of AKI (Fig. 7). Inhibition of PANX1 channels could therefore be a therapeutic approach to reduce renal cells apoptosis and to maintain healthy mitochondrial population to combat AKI development.

## Methods

### Study design and participants

This study was a prospective observational study. Approved by the ethics committee of Central South Hospital of Wuhan University in accordance with the principles of the Declaration of Helsinki, the patients who were transferred to the intensive care unit after cardiopulmonary bypass surgery in Zhongnan Hospital of Wuhan University from 1 January to 30 June 2019 were included in this study. Patients aged under 18 years or over 80 years were excluded from the study. Patients who had AKI, chronic kidney disease, end-stage renal disease, or tumor, had received renal replacement therapy, and pregnant patients were excluded from the study. Informed consent was obtained from the patients or their guardians. Patients who did not fill in the consent form were also excluded.

### Sample collection and serum PANX1 measurement

When the patient was sent to the intensive care unit after cardiopulmonary bypass, blood samples were collected, centrifuged at 1500 × *g* for 10 minutes, and the serum was collected and stored at −80 °C for analysis. At the same time, the relevant information of the patients was recorded.

The serum PANX1 concentration was quantified using the commercially available enzyme-linked immunosorbent assay kit (Jymbio, Colorful Gene Biological Technology Co. Ltd., Wuhan, China) according to the manufacturer’s protocol.

### Animals

C57BL/6 J wild-type mice and PANX1−/− mice were used in the study. PANX1−/− mice were purchased from Gem Pharmatech Co. Ltd. (Nanjing, China), and were created from C57BL/6 J wild-type mice and PANX1+/− mice. All mice used for experiments were 8–10 weeks old males and were raised under specific pathogen-free and controlled temperature conditions with a 12 h light/dark cycle. All animal experiments were approved by The Animal Care and Use Committee of Zhongnan Hospital of Wuhan University. All animal experimental procedures were performed in accordance with national and EU guidelines. Additionally, all animals were provided humane care according to the ARRIVE guidelines.

### Kidney I/R mouse model

The model of kidney ischemia in mice established in this study is stable according to previous studies^19^. For kidney I/R surgery, mice were anesthetized the abdomen along the midabdominal line was opened, and the bilateral renal pedicles were occluded using a microvascular clamp. The clamp was removed after 30 min of ischemia to initiate reperfusion. All kidney tissues and serum were then collected for analysis. In the sham group, mice were subjected to the same process without clamping of the kidney.

### Serum chemistry assays

To detect the level of serum creatinine, heparinized blood was centrifuged at 2000 rpm for 10 min to acquire the plasma. The level of creatinine was detected by the ADVIA 2400 Chemistry System (Siemens, Tarrytown, NY, USA) according to the manufacturer’s protocols.

### Histopathology and TUNEL assay

Kidney tissues were fixed with 4% paraformaldehyde, embedded 10% in paraffin, and sectioned at 4 μm for hematoxylin and eosin (HE) staining. For the detection of TUNEL-positive cells, the ApopTag Peroxidase In Situ Apoptosis Detection Kit was used according to the manufacturer’s instructions (S7100, Serologicals, Millipore).

### DHE staining

The kidneys were rinsed in pre-cooled 4 °C PBS after nephrectomy. The kidney tissue was placed in the frozen section embedding agent (Yeasen) and rapidly frozen in liquid nitrogen. 20 μM thick frozen kidney tissue sections were then incubated at 37 °C with 10 μM DHE for 30 min, and the renal tissue superoxide was analyzed by confocal microscopy.

### Cell culture

HK2 cells were purchased from the Cell Bank of the Chinese Academy of Sciences. The cells were cultured in minimum essential medium (HyClone) with 5 ng/ml human recombinant epidermal growth factor (Novus), 10% fetal bovine serum (Gibco), and a penicillin-streptomycin supplement. The cells were maintained in a humidified incubator at 37 °C and 5% CO_2_.

### Cells hypoxia/reoxygenation model

The HK2 cells were seeded and cultured overnight in complete MEM. After the medium was changed to serum and glucose-free MEM, cellular hypoxic conditions were created in a modular incubator chamber (Biospherix, Lacona, NY, USA) and maintained by continuous gas flow with a 1% O_2_, 5% CO_2_, and 94% N2 gas mixture. Cells were incubated under normal conditions (95% air and 5% CO_2_) for 60 minutes after the indicated times of H/R.

### Plasmids, short interfering RNA, and transfection

The mt-Keima-cox8 plasmid (#206907, Addgene) and empty ctrl plasmid (#206908, Addgene) was obtained from Public Protein/Plasmid Library (Nangjing, China). Human PANX1-shRNA, PANX1-shctrlRNA, overexpression plasmids (#206909, Addgene) and PANX1 empty ctrl plasmid (#206910, Addgene) were purchased from GenePharma (Shanghai, China). Short interfering RNA (siRNA) oligonucleotides against P2Y2 was synthesized by Public Protein/Plasmid Library (Nangjing, China). Short interfering RNA (siRNA) oligonucleotides against ATG13 were synthesized by Cohesion Biosciences (Suzhou, China).

### Materials

The following compounds were obtained from the sources indicated and used at the final concentrations indicated: ATP (100 µM, Sigma-Aldrich), suramin (100 µM, Sigma-Aldrich), Diquafosol (10 µM, MedChemExpress), rapamycin (0.1 µM, Sigma-Aldrich), MHY1485 (10 µM, MedChemExpress).

### Assessment of intracellular and extracellular ATP content

ATP content was measured by a luciferin-luciferase bioluminescence assay. To measure intracellular ATP content. Cells were extracted with Reporter lysis buffer, the supernatants were collected for ATP measurement using an ATP determination kit as described by the manufacturer (S0027, Beyotime). To measure extracellular ATP content, we used the Enliten ATP Assay Kit (FF2000, Promega, Madison, WI) and followed the manufacturer’s instructions. For each measurement, a standard curve was constructed using ATP standard solutions.

### Measurement of intracellular mitochondrial ROS

MitoSOX Red Mitochondrial Superoxide Indicator (Thermo Fisher Scientific, M36008) was used to measure mitochondrial superoxide, following the manufacturer’s instructions. In brief, after the aforementioned treatments, cells were collected and washed three times with 1× PBS, followed by incubation with 5 µM MitoSOX Red for 45 min at 37 °C in the dark. Luminescence was then measured.

### Flow cytometry

After treatment, 10^6 cells were collected, centrifuged, and discarded the supernatant. Cell death was detected by a commercialized apoptosis detection kit (C1062M, Beyotime). MMP was detected by a commercialized JC-1 kit (J8030, Solarbio). Mitochondrial ROS was detected by a commercialized ROS kit (40778ES50, Yeasen). Fluorescence was analyzed using a flow cytometer. Single parameter histograms were applied to density plots to exclude debris. Unstained control and single color stained controls, were used to defend boundaries between positive and negative staining cell populations.

### Transmission electron microscopy

Kidneys were prefixed in 2% glutaraldehyde, and fixed in 1% osmium tetroxide. Next, samples were dehydrated in ethanol with 3% uranyl acetate, embedded in the epoxy resin and propylene oxide overnight, and polymerized. After slicing into 70-nm-thick sections and staining with lead citrate, the sections were detected by transmission electron microscope.

### Western blotting analyses and quantitative RT-PCR

Total protein was extracted from HK2 cells and mice kidney tissues. Protein concentrations were determined by Coomassie Brilliant Blue G-250 (Bio-Rad, Hercules, CA, USA). Each sample well was loaded with 10 µl of a 2 mg/ml final concentration of denatured protein. Equal amounts of proteins were separated by SDS-PAGE and transferred to a nitrocellulose membrane (Millipore). The membrane was incubated with specific antibody. All antibodies used for western blotting analyses are described in Supplementary Table 1. ImageJ was used to quantify the protein expression, and GAPDH served as the control. In addition, mRNA RT was performed with the ReverTra Ace kit (Toyobo, Osaka, Japan). The cDNA then served as the template for SYBR real-time PCR. All reactions were run in triplicate on the Real-Time PCR Detection System (Bio-Rad). All primer sequences used for RT-PCR analyses are described in Supplementary Table 2.

### Immunohistochemical and immunofluorescence staining

Paraffin-embedded kidney sections (4 μm) were deparaffinized, and ethylene diamine tetra acetic acid (1 mM) was used for antigen retrieval. Hydrogen peroxide (H2O2, 0.03%) was used for the immunohistochemical study, and the primary antibody was incubated overnight at 4 °C. For immunofluorescence, the slides were incubated with primary antibodies at 4 °C overnight. The images were detected by fluorescence microscopy, and positive images were analyzed by computerized digital image analysis. The intensity of immunofluorescence was analyzed using Image J.

HK2 cells were cultivated on cell slides for staining. 0.1% Triton-X-100 was used to permeate cell membrane for 5 min at room temperature. The cell slides were incubated with primary antibodies at 4 °C overnight. After incubating with a secondary antibody, the images were detected by confocal microscopy or fluorescence microscopy.

### Statistics and reproducibility

At least three individual experiments were performed in all the studies unless stated otherwise. All replicates indicated in legends are biological replicates. All numerical data was expressed as mean ± standard error or median and interquartile range. Student’s *t* test, One-way ANOVA with Dunnett’s multiple comparisons test, and Mann–Whitney *U* tests were used to compare the continuous variables. Statistical analyses were performed using Statistical Package for the Social Sciences (version 22.0), and GraphPad Prism software (version 8.0). *P* < 0.05 was considered to be statistically significant.

### Reporting summary

Further information on research design is available in the Nature Portfolio Reporting Summary linked to this article.

### Supplementary information

Supplementary information

Description of Additional Supplementary Files

Supplementary Data

Reporting Summary

## Data Availability

Supplementary Tables 1, 2, and Supplementary Figs. 1,2 have been provided in the supplemental information pdf file. Supplementary Fig. 3 in the supplemental information pdf file provides a gating strategy. Supplementary Fig. 4 in the supplemental information pdf file provides all original western blot images with size markers. Numerical source data for Figs. 1–6 and Supplementary Figs. 1, 2 have been provided in Supplementary Data. Plasmids in our study were deposited in Addgene. All other data are available from the corresponding author (or other sources, as applicable) on reasonable request.
