# Supplementary material for: Pannexin 1 targets mitophagy to mediate renal ischemia/reperfusion injury
Source: Commun Biol. 2023 Aug 29;6:889. doi: 10.1038/s42003-023-05226-x (PMC10465551; doi:10.1038/s42003-023-05226-x)

Pannexin 1 targets mitophagy to mediate renal ischemia/reperfusion injury

Lianjiu Su[1, 2, 3#*], Jiahao Zhang[1#], Jing Wang[1, 2#], Xiaozhan Wang[1, 2#], Edward Cao[3], Chen Yang[1], Qihao Sun [3], Ramadoss Sivakumar[3], Zhiyong Peng[1, 2, 4*]

[1]Department of Critical Care Medicine, Zhongnan Hospital of Wuhan University, Wuhan 430071, Hubei, China.

[2]Clinical Research Center of Hubei Critical Care Medicine, Wuhan, China.

[3]Department of Cardiology, David Geffen School of Medicine, University of California, Los Angeles, California, USA

[4]Center of Critical Care Nephrology, Department of Critical Care Medicine, University of Pittsburgh Medical Center, Pittsburgh, PA 15206

# Authors contributed equally to this study and therefore shared the first author role

* Correspondence to: Zhiyong Peng, MD, PhD. E-mail: pengzy5@hotmail.com and Lianjiu Su, MD, PhD. E-mail: sulianjiu@whu.edu.cn. Department of Critical Care Medicine Zhongnan Hospital of Wuhan University 169 Donghu Rd, Wuchang District Wuhan 430071, Hubei province, China.

Supplementary Table 1: Antibodies for immunoblot analyses.

Antibody	Cat No.	Manufacturer	species	dilution
PANX1	12595-1-AP	Proteintech	rabbit	1:1000
PANX1	#91137	Cell Signaling Technology	rabbit	1:1000
PANX1	sc-293210	Santa Cruz	mouse	1:200
GAPDH	60004-1-Ig	Proteintech	mouse	1:50000
LC3B	18725-1-AP	Proteintech	rabbit	1:500
LC3	14600-1-AP	Proteintech	rabbit	1:1000
P62	18420-1-AP	Proteintech	rabbit	1:5000
TOM20	11802-1-AP	Proteintech	rabbit	1:2000
TIM23	67535-1-Ig	Proteintech	mouse	1:2000
PINK	DF7742	Affinity	rabbit	1:1000
PARKIN	AF0235	Affinity	rabbit	1:1000
Caspase 3	19677-1-AP	Proteintech	rabbit	1:1000
Bax	50599-2-Ig	Proteintech	rabbit	1:1000
Bcl2	26593-1-AP	Proteintech	rabbit	1:1000
mTOR	#2972	Cell Signaling Technology	rabbit	1:1000
Phospho-mTOR (Ser2448)	#5536	Cell Signaling Technology	rabbit	1:1000
Phospho-mTOR (Ser2481)	#2974	Cell Signaling Technology	rabbit	1:1000
Phospho-p70 S6 Kinase	AF3228	Affinity	rabbit	1:1000
Phospho-ULK1	AF4387	Affinity	rabbit	1:1000
Phospho-4E-BP1	AF3432	Affinity	rabbit	1:1000
p70 S6 Kinase	AF6226	Affinity	rabbit	1:1000
ULK1	AF7588	Affinity	rabbit	1:2000
4E-BP1	AF4387	Affinity	rabbit	1:1000

Supplementary Table 2: Primers for Real-time PCR detection.

**Gene**		**Sequence5'---3'**
PANX1(mouse)	F	CTTCACTCTCGGACGAGTTTC
PANX1(mouse)	R	GGAATCAGCAGAGCATACACAAT
PANX1(human)	F	GCTCTTTGCGATCCTCCTGTA
PANX1(human)	R	TGCACGGTTGTAAACTTTGTCAA
GAPDH (mouse)	F	AGGTCGGTGTGAACGGATTTG
GAPDH (mouse)	R	GGGGTCGTTGATGGCAACA
GAPDH (human)	F	GAAGGTGAAGGTCGGAGTC
GAPDH (human)	R	GAAGATGGTGATGGGATTTC
P2Y2 (human)	F	CGTGGCCTACAGCTCAGTC
P2Y2 (human)	R	GTGACGTGGAATGGCAGGAA
ATG13 (human)	F	TAGGCGGCATGCGAGGCTACGC
ATG13(human)	R	ATCGGCTAGCTAGCTACGATA
Kim-1(mouse)	F	AAACCAGAGATTCCCACACG
Kim-1(mouse)	R	GTCGTGGGTCTTCCTGTAGC
NGAL (mouse)	F	CCATCTATGAGCTACAAGAGAACAAT
NGAL (mouse)	R	TCTGATCCAGTAGCGACAGC

Supplementary Figure 1

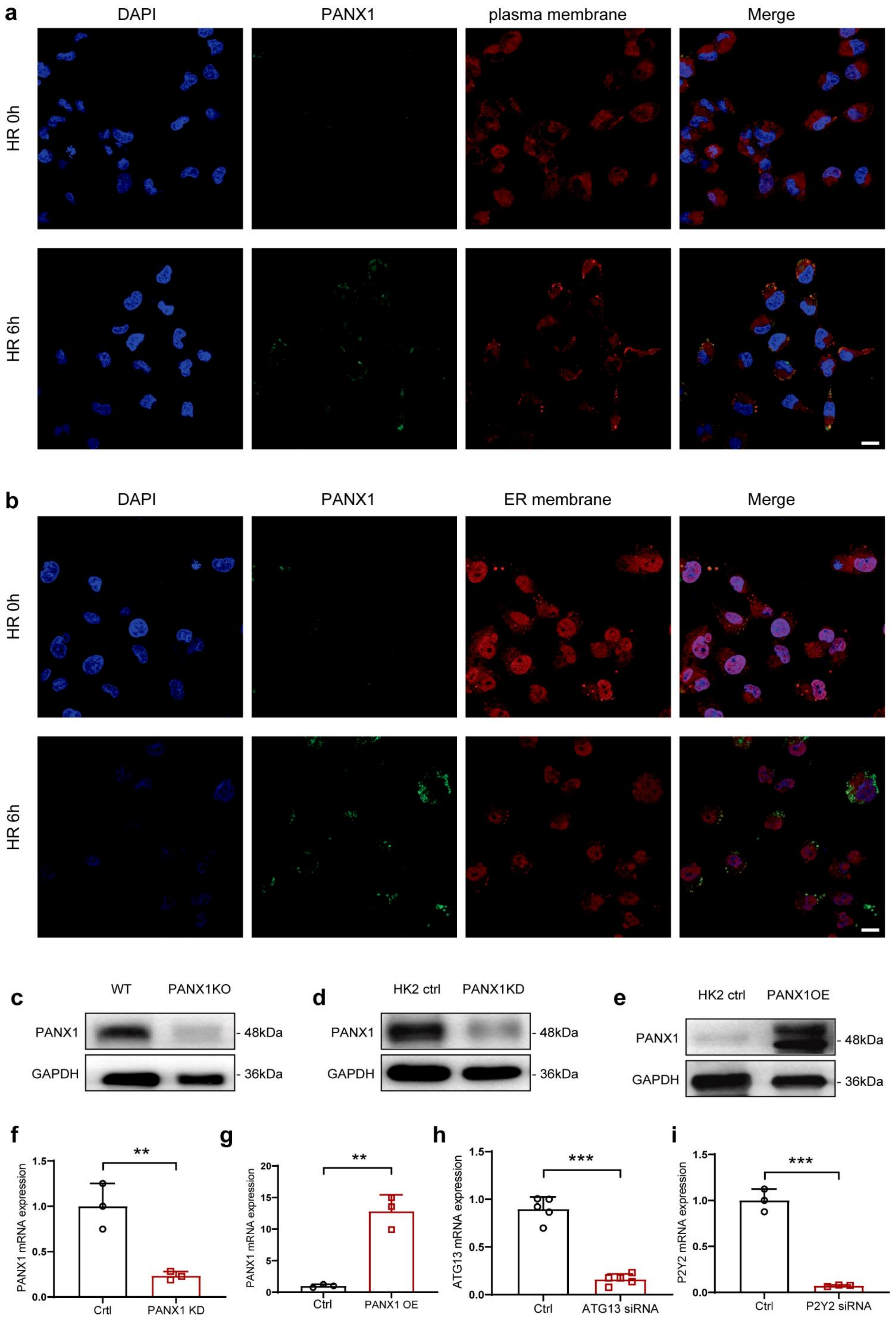

Supplementary Figure 1

Supplementary Figure 1: (**a**) Laser confocal scanning microscopy for PANX1and plasma membrane staining (Bar=20μm). (**b**) Laser confocal scanning microscopy for PANX1 and endoplasmic reticulum staining (Bar=20μm). (**c**) Western blot for PANX1 in WT and PANX1-/- mice. (**d**) Western blot for PANX1 in HK2 ctrl and PANX1KD. (**e**) Western blot PANX1 in HK2 empty and PANX1OE. (**f**) qRT-PCR for PANX1 in HK2 ctrl and PANX1KD (n=3). (**g**) qRT-PCR for PANX1 in HK2 empty and PANX1OE (n=3). (**h**) qRT-PCR for ATG13 in HK2 ctrl and ATG13 siRNA (n=5). (**i**) qRT-PCR for P2Y2 in HK2 ctrl and P2Y2 siRNA (n=5). GAPDH served as the loading control. Results are presented as means ± SEM. P values from Student's t test. * indicates $p<0.05$; ** indicates $p<0.01$; *** indicates $p<0.001$.

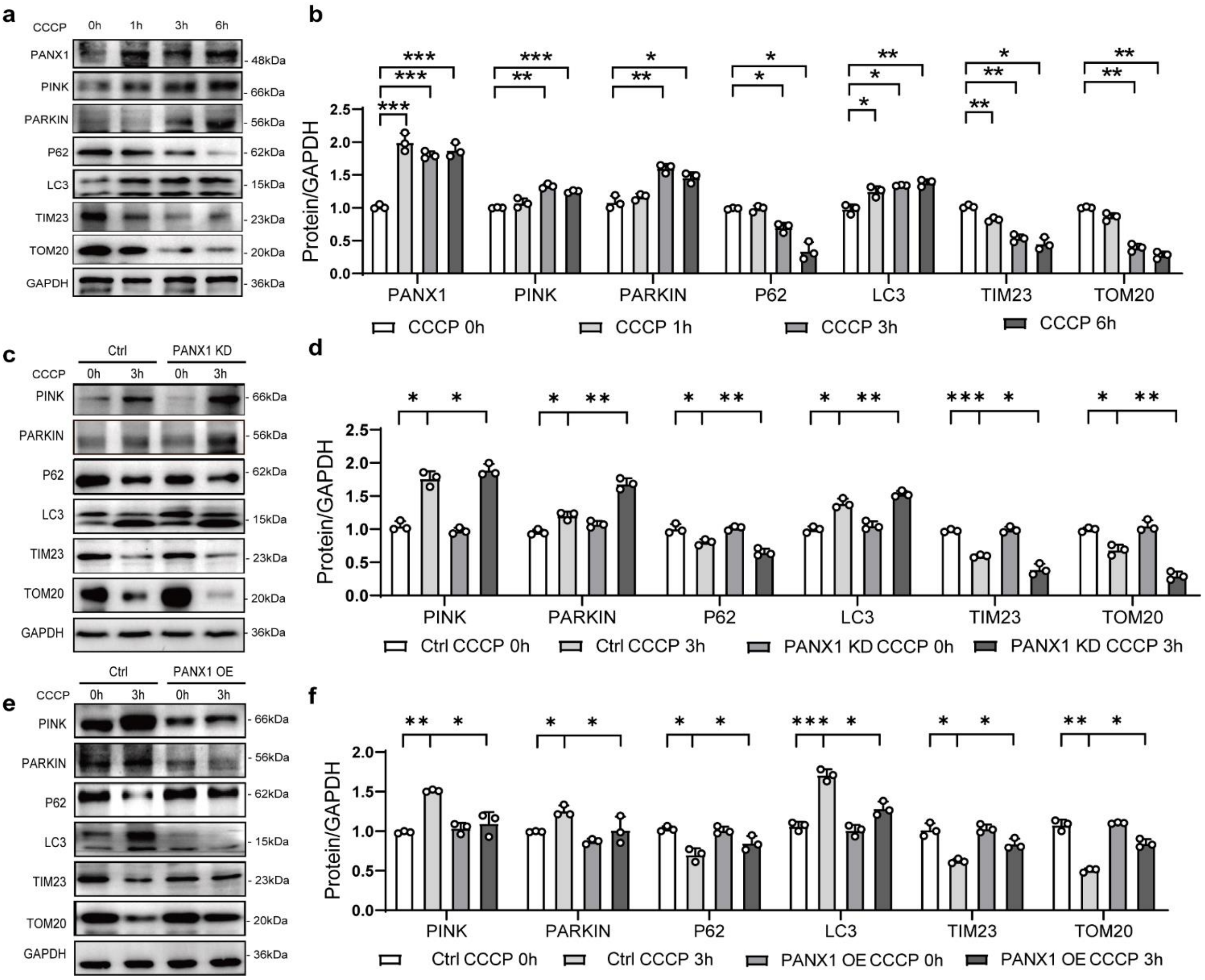

Supplemental Fig. 2. (**a**, **b**) Western blot measured the protein levels of mitophagy-related proteins in HK2 cells subjected to CCCP for the indicated time points (n=3). Results are presented as means ± SEM. P values from One-way ANOVA with Dunnett's multiple comparisons test. (**c-f**) Western blot indicated the levels of mitophagy-related proteins in PANX1KD and PANX1OE cells treated with CCCP (n=3). Results are presented as means ± SEM. P values from Student's t test. GAPDH served as the loading control. * indicates p<0.05; ** indicates p<0.01; *** indicates p<0.001.

Supplementary Figure 3. Gating strategy

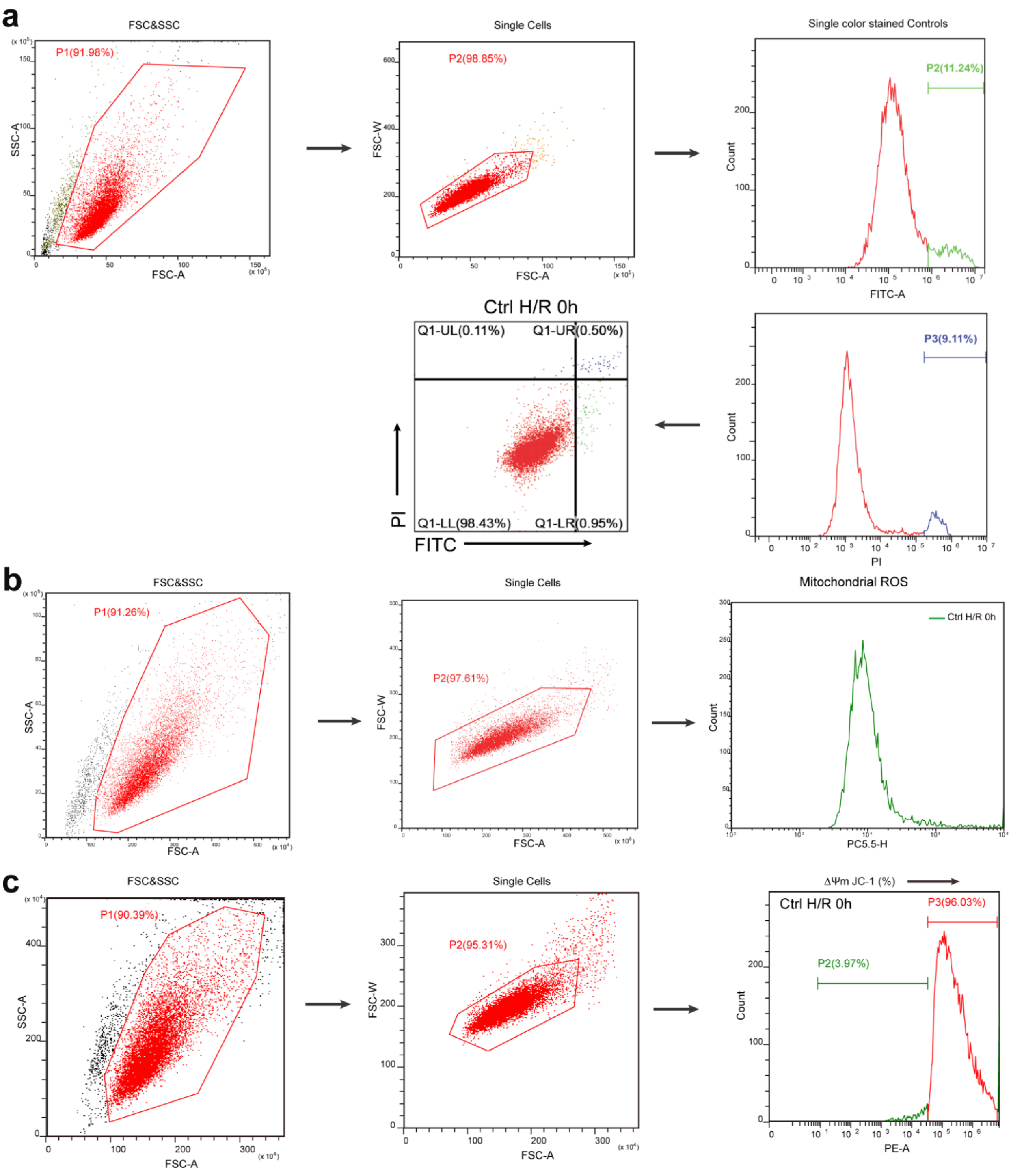

Supplemental Fig. 3. (**a**) Gating strategy for apoptotic cell death in Fig. 3h. (**b**) Gating strategy for mitochondrial ROS in Fig. 4b. (**b**) Gating strategy for JC-1 in Fig. 4d.

Supplementary Figure 4. Original blot images

Figure 2a

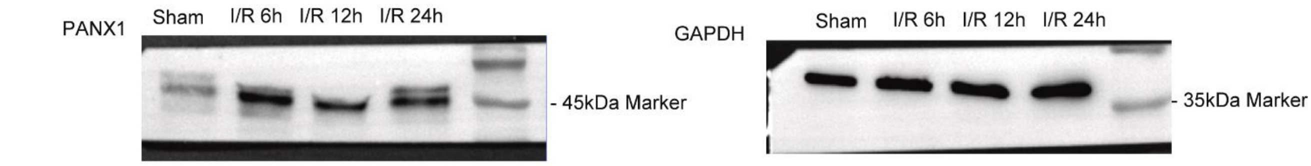

Figure 2d

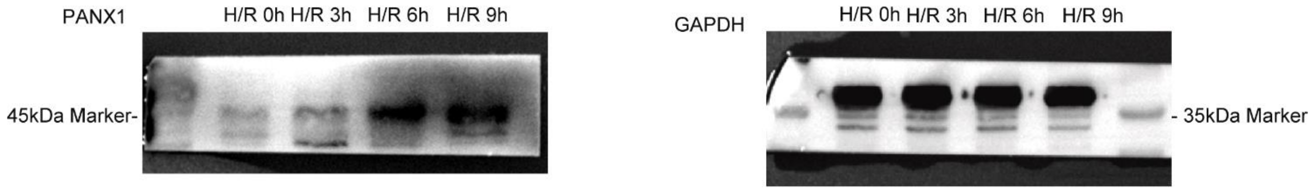

Figure 3f

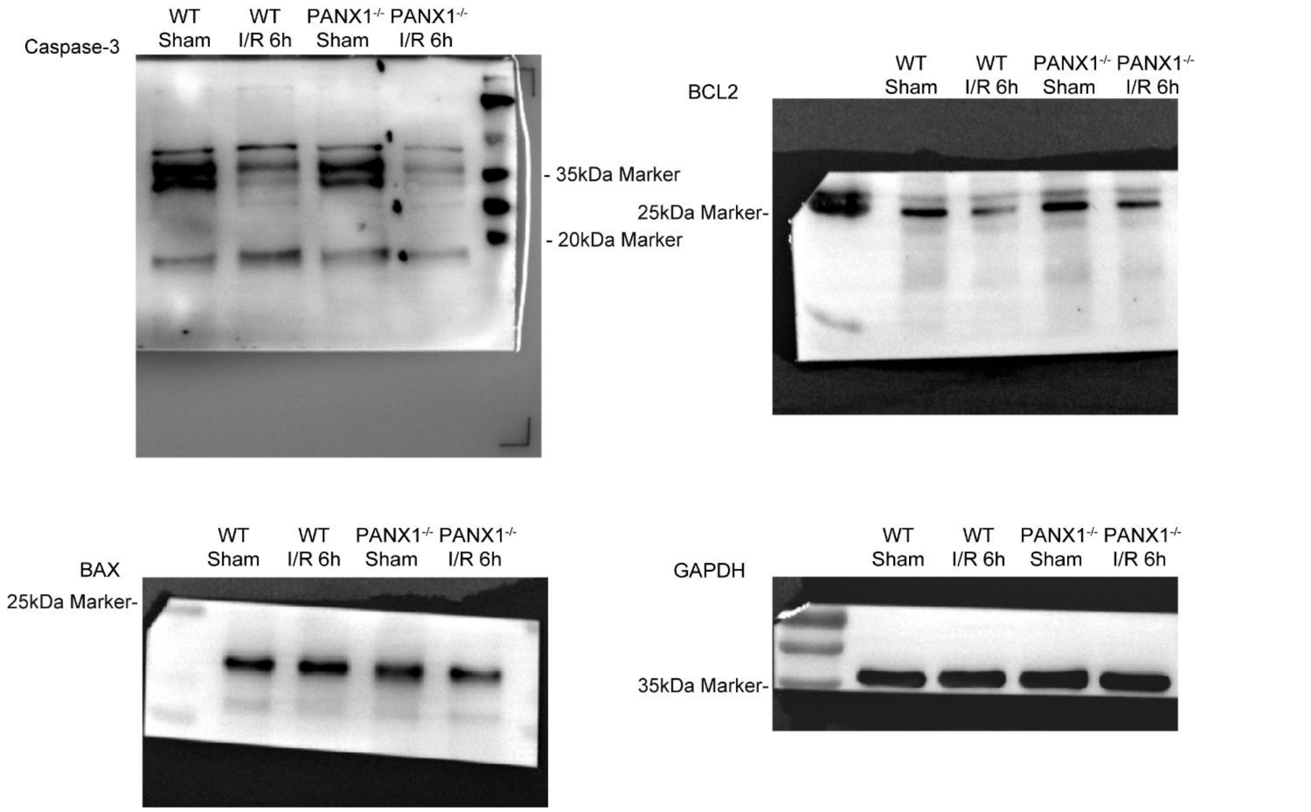

Supplementary Figure 4. Original blot images

Figure 3j

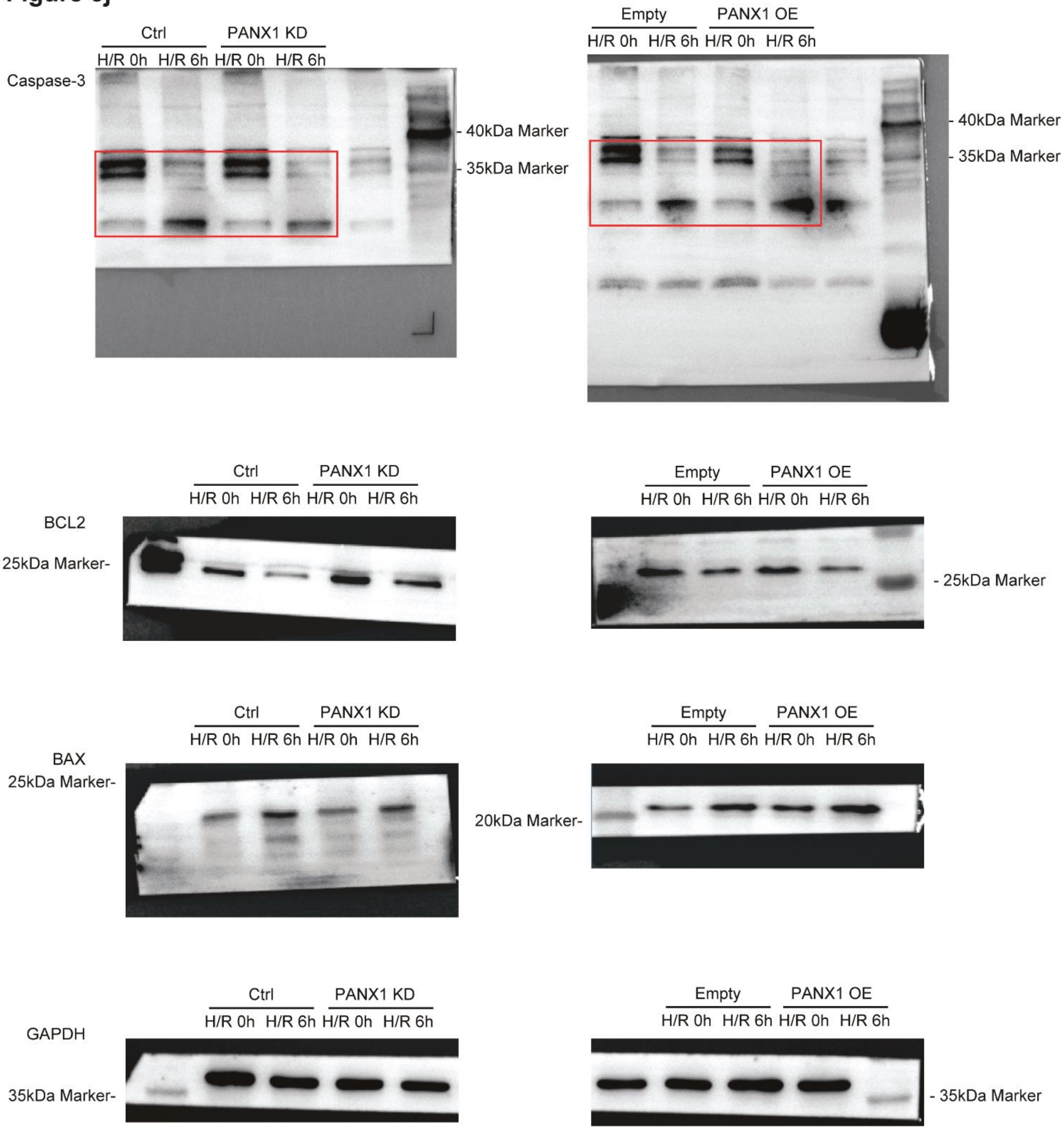

Figure 5a

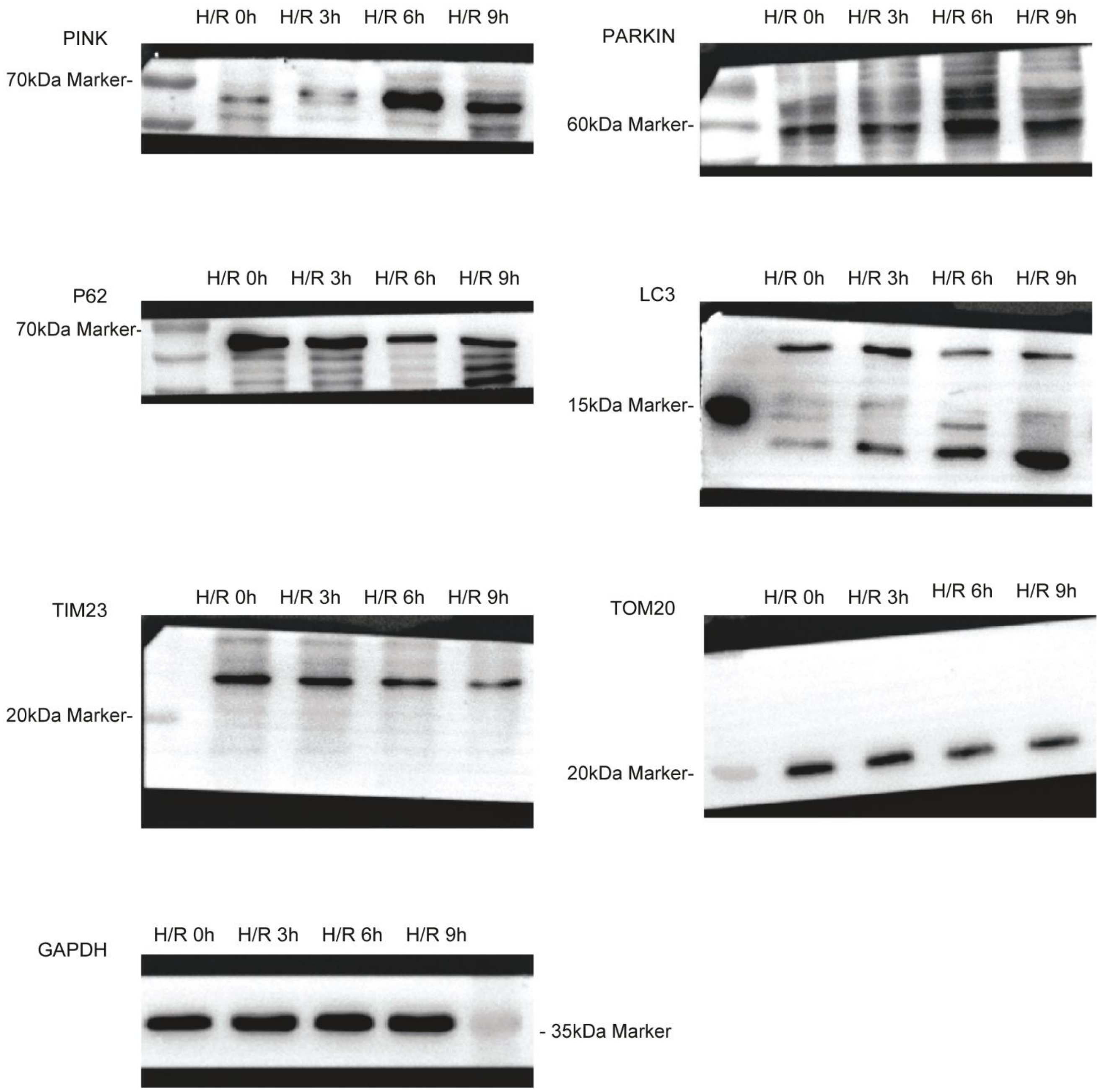
PINK
H/R 0h
H/R 3h
H/R 6h
H/R 9h
70kDa Marker-
PARKIN
H/R 0h
H/R 3h
H/R 6h
H/R 9h
60kDa Marker-
P62
H/R 0h
H/R 3h
H/R 6h
H/R 9h
70kDa Marker-
LC3
H/R 0h
H/R 3h
H/R 6h
H/R 9h
15kDa Marker-
TIM23
H/R 0h
H/R 3h
H/R 6h
H/R 9h
20kDa Marker-
TOM20
H/R 0h
H/R 3h
H/R 6h
H/R 9h
20kDa Marker-
GAPDH
H/R 0h
H/R 3h
H/R 6h
H/R 9h
- 35kDa Marker

Supplementary Figure 4. Original blot images

Figure 5e

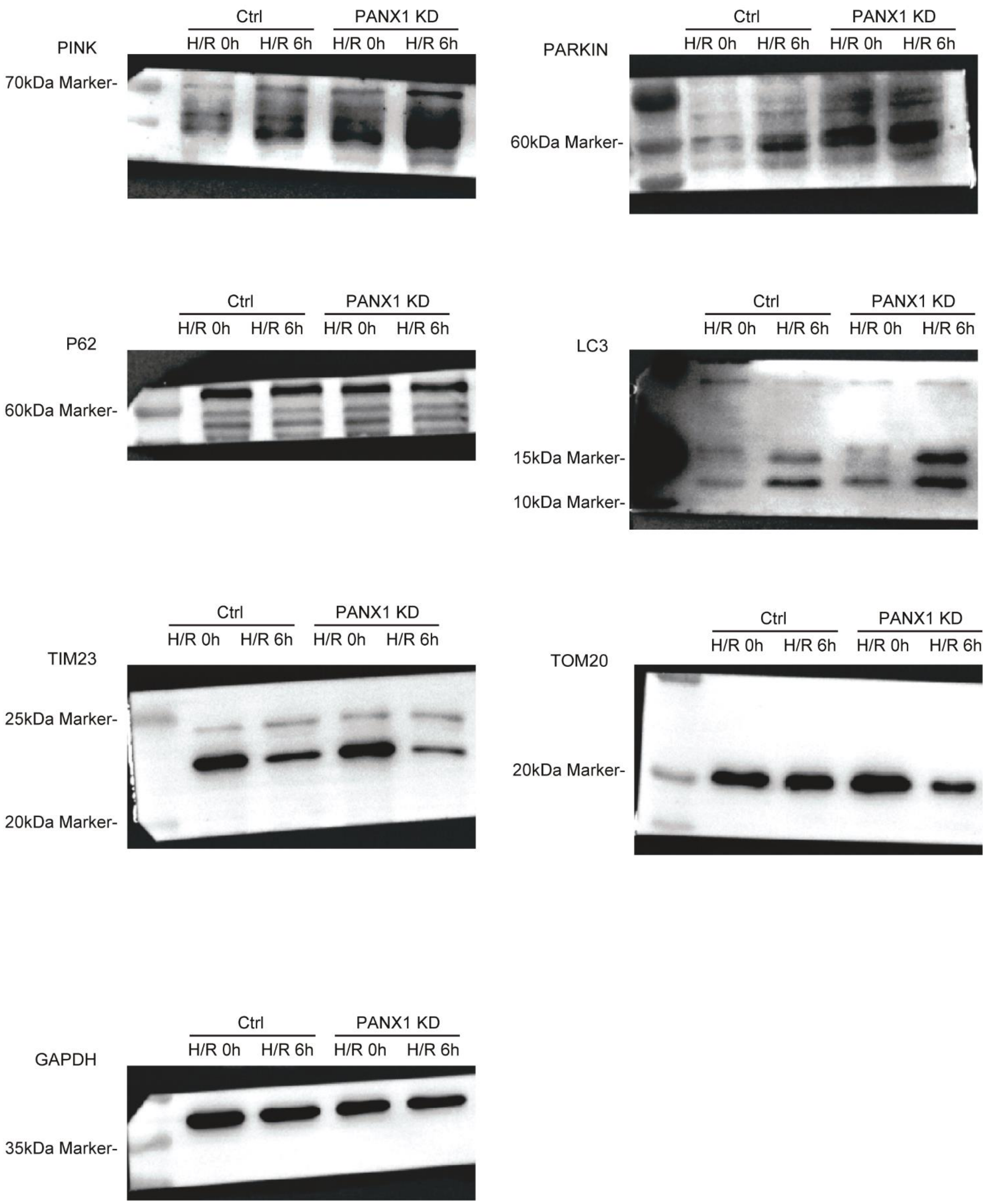

Supplementary Figure 4. Original blot images

Figure 5g

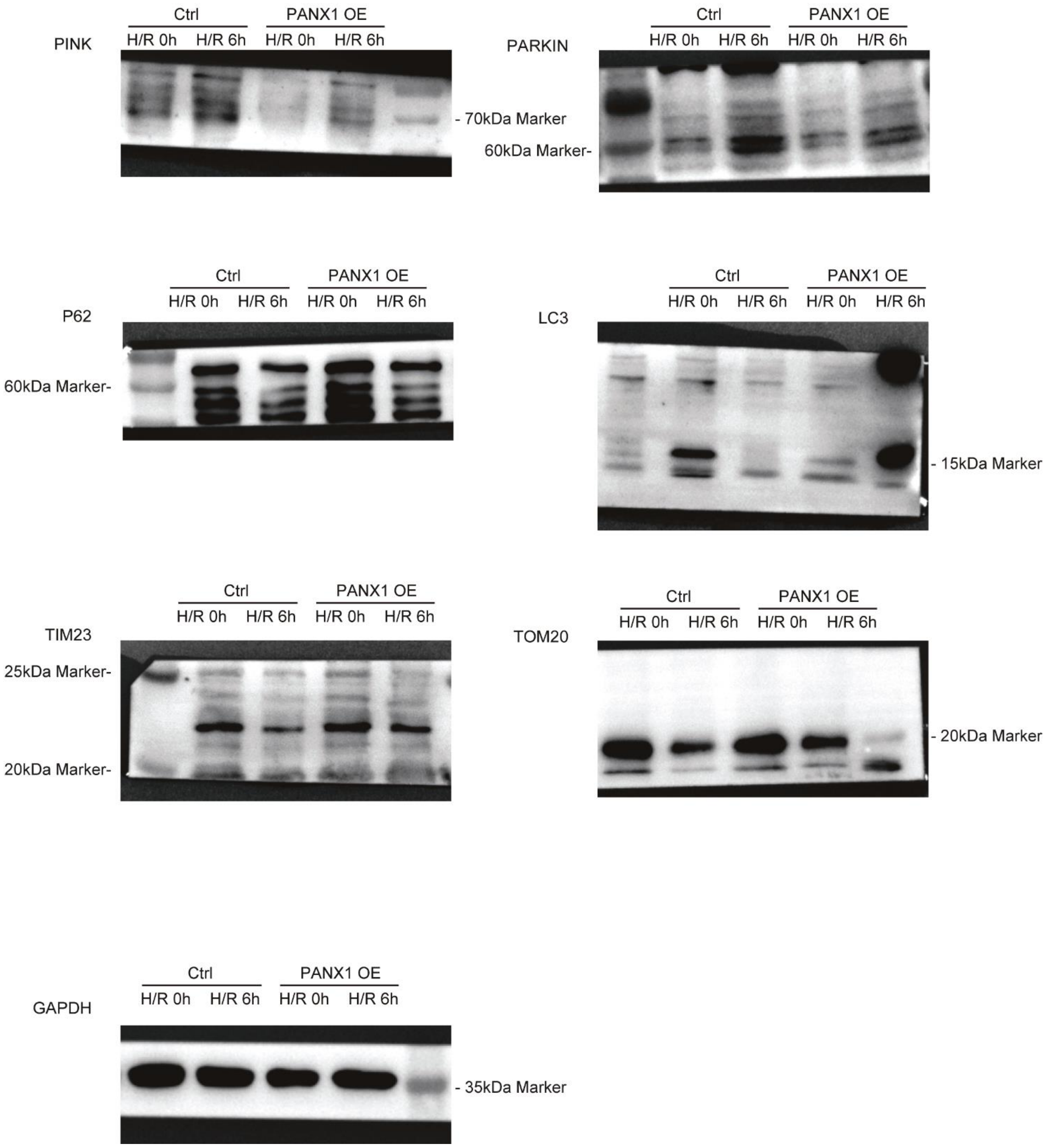

Supplementary Figure 4. Original blot images

Figure 5k

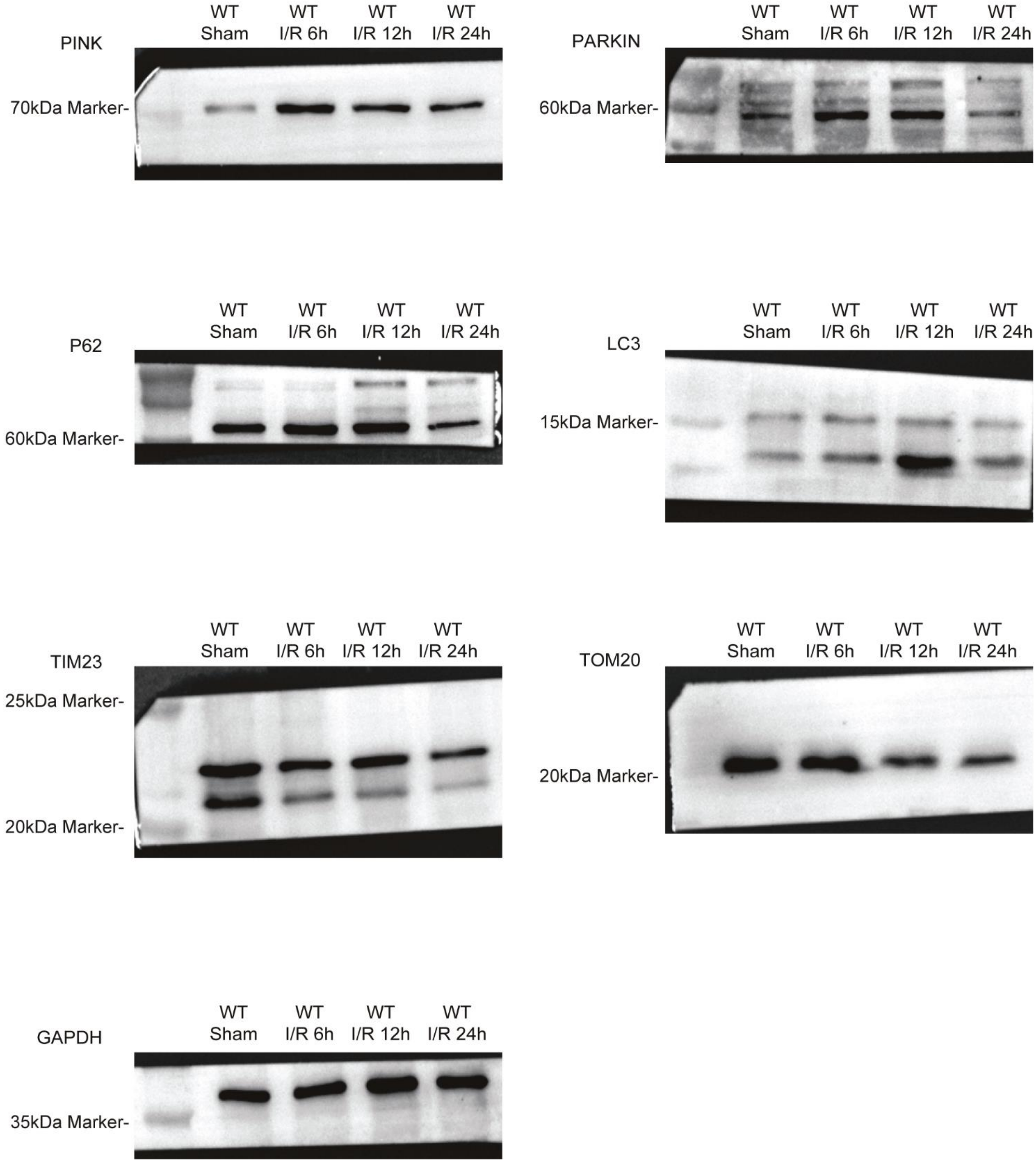

Supplementary Figure 4. Original blot images

Figure 5m

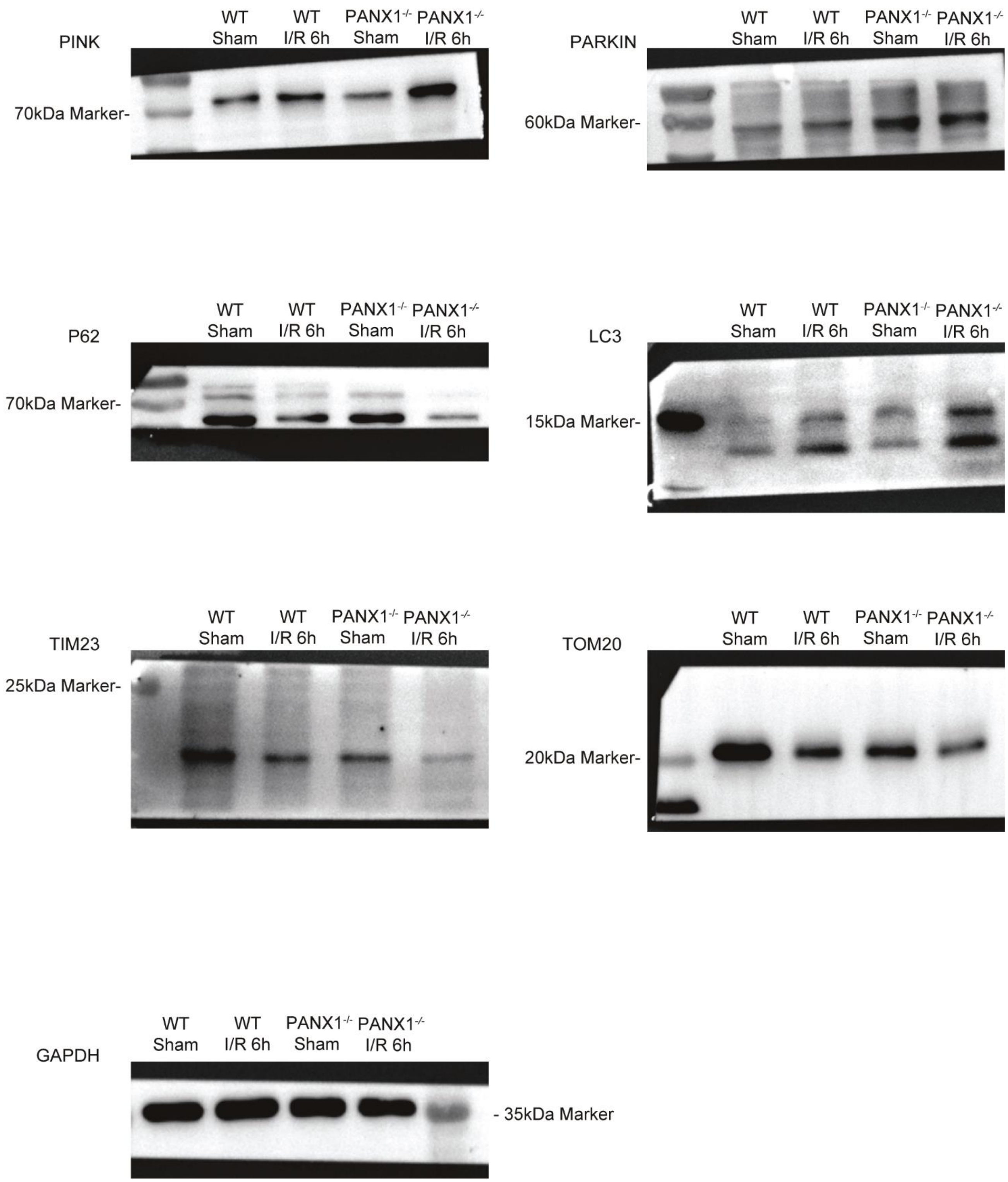

Supplementary Figure 4. Original blot images

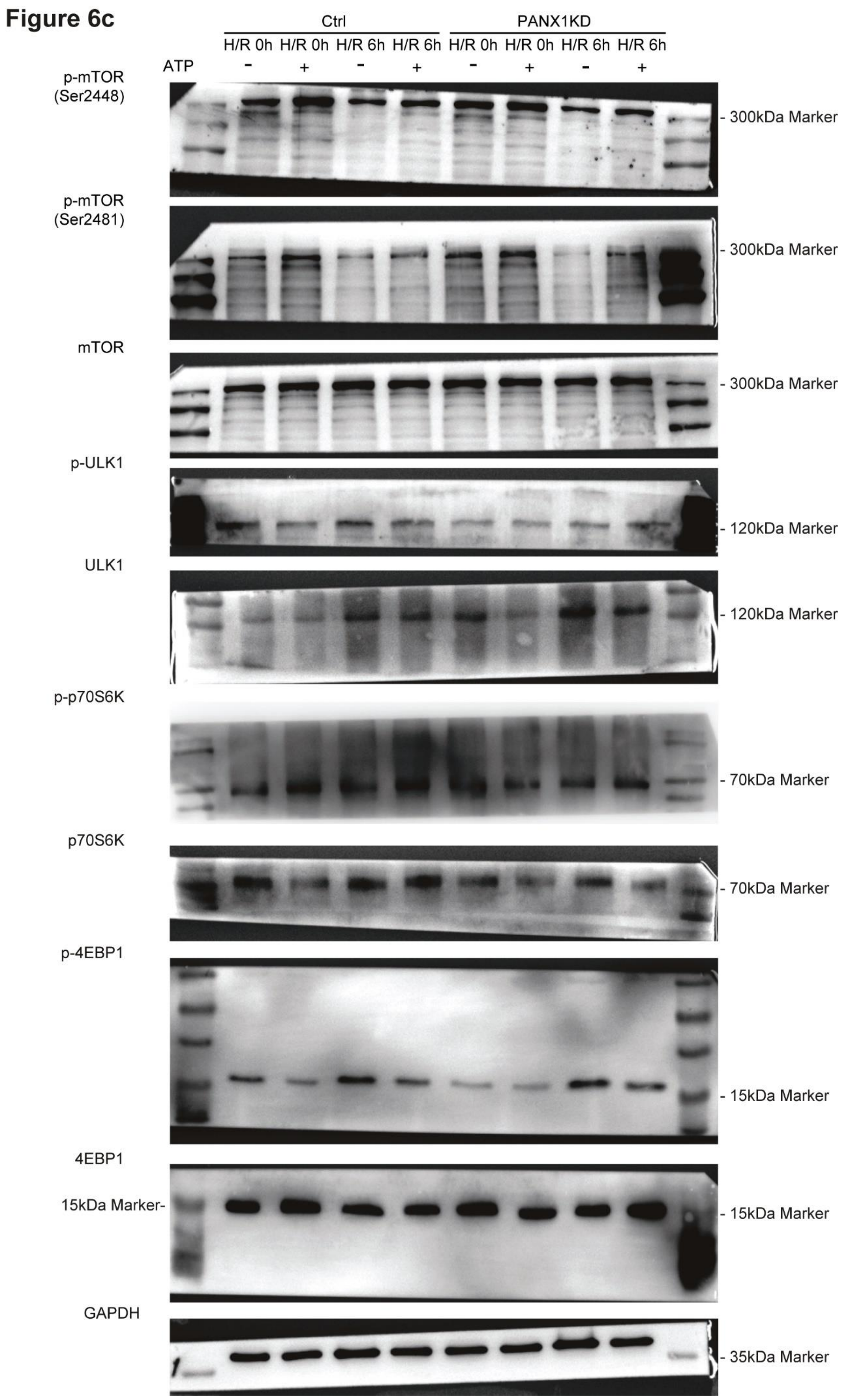

Supplementary Figure 4. Original blot images

Figure 6e

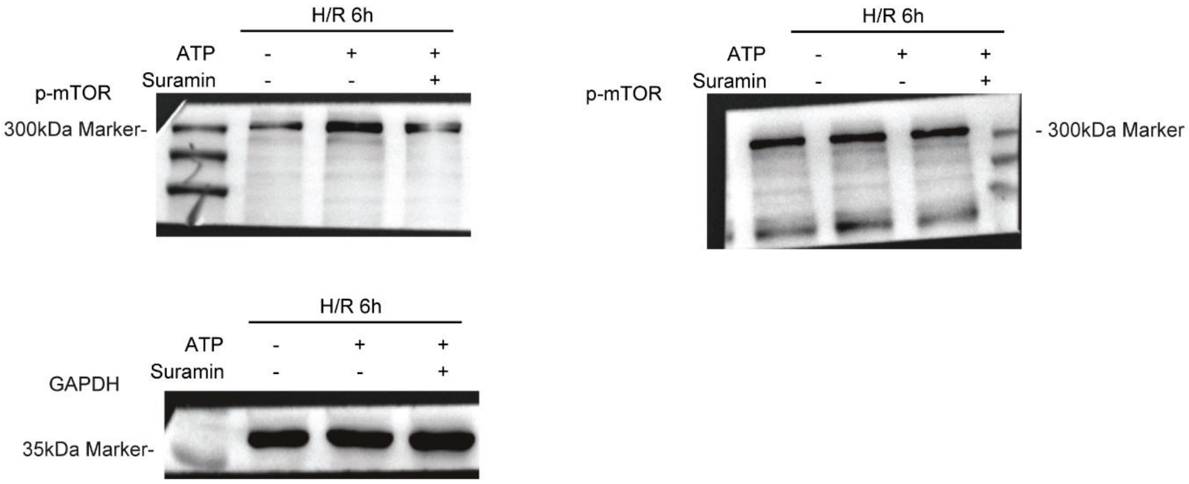

Figure 6g

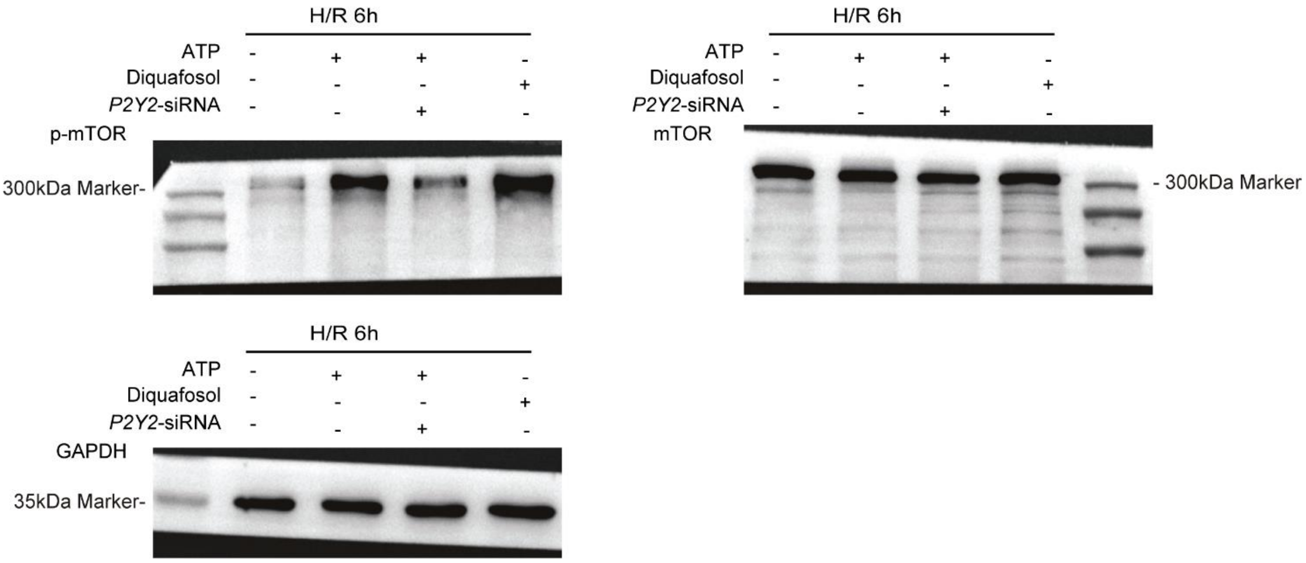

Supplementary Figure 4. Original blot images

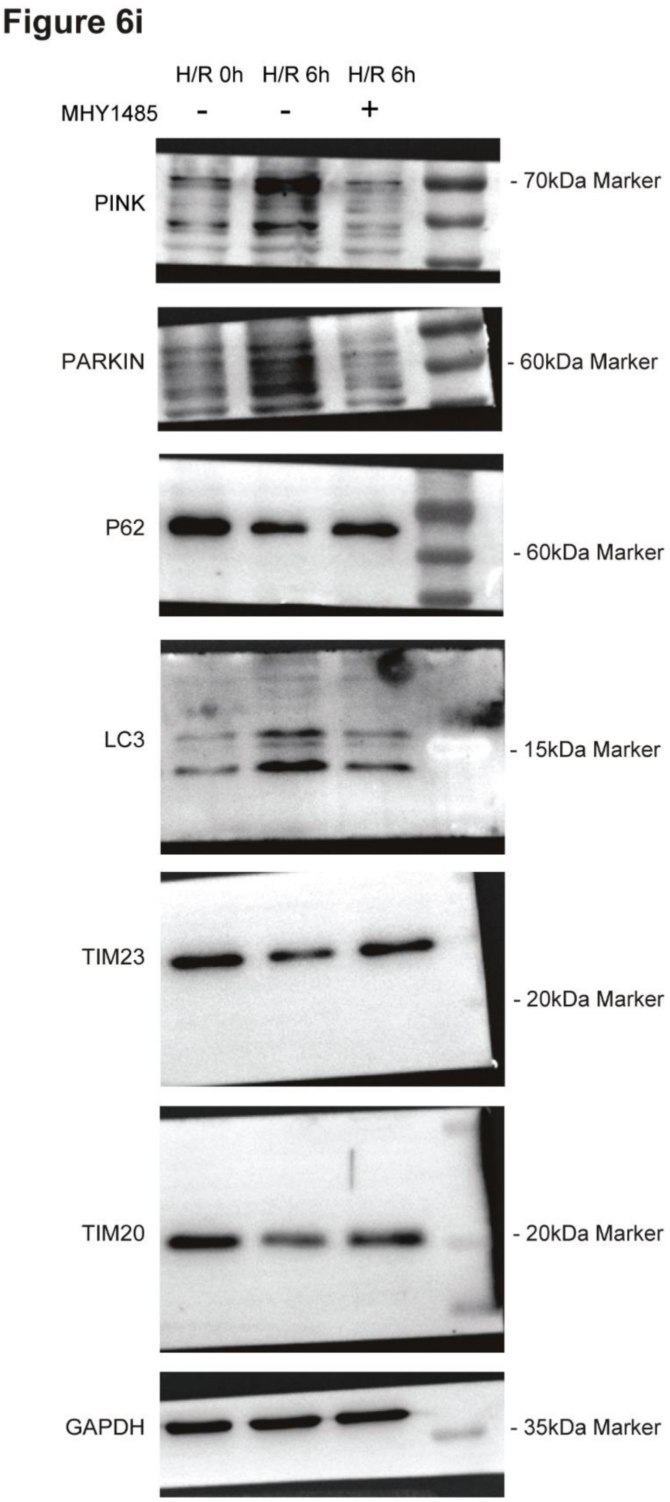

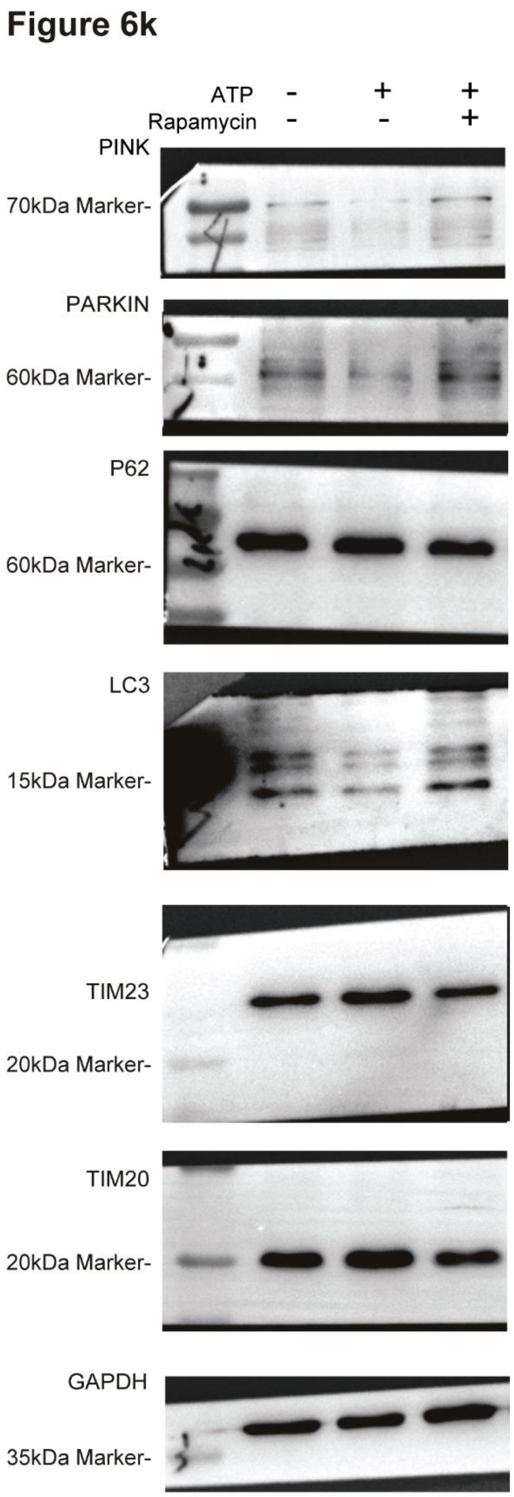

Supplementary Figure 4. Original blot images

Supplementary Figure 1c

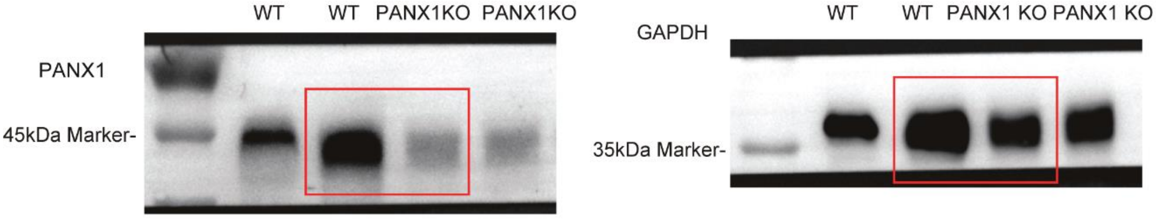

Supplementary Figure 1d

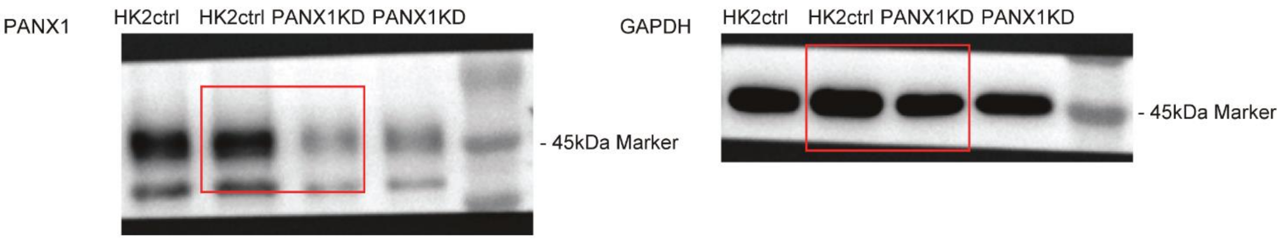

Supplementary Figure 1e

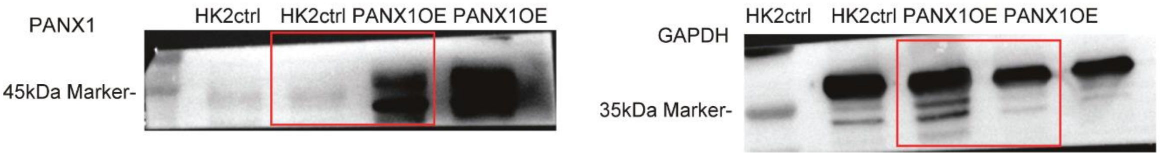

Supplementary Figure 4. Original blot images

Supplementary Figure 2a

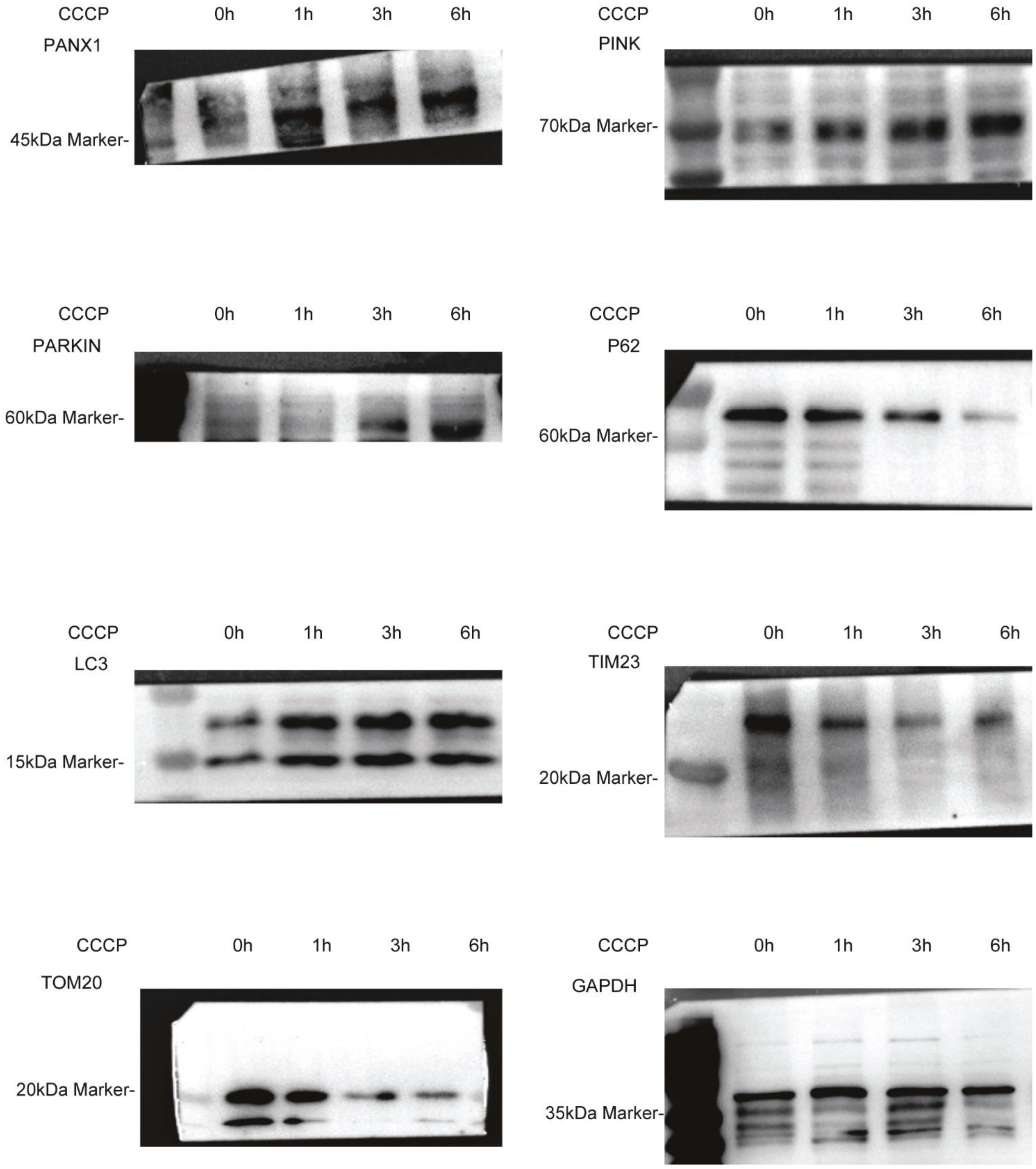

Supplementary Figure 4. Original blot images

Supplementary Figure 2c

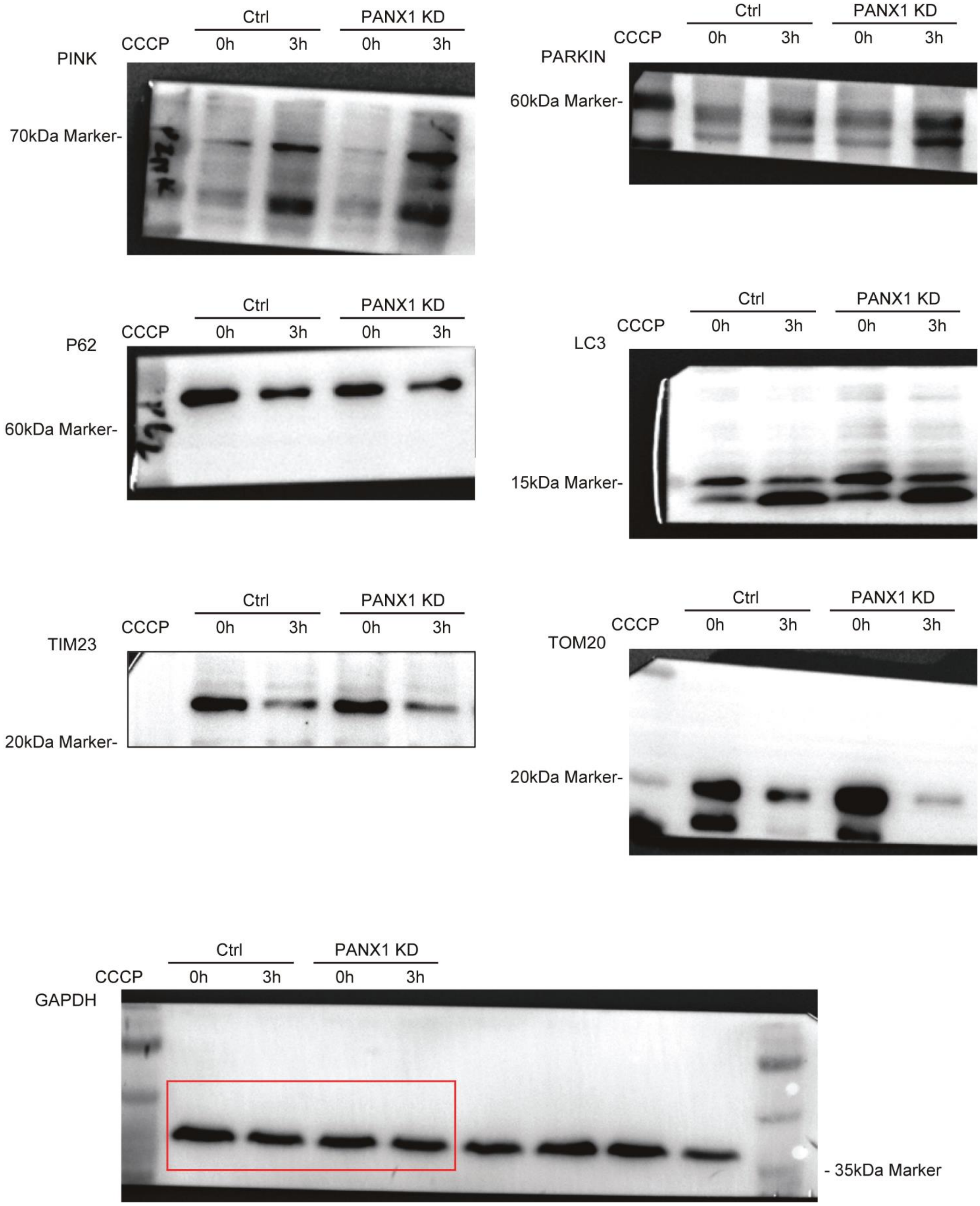

Supplementary Figure 4. Original blot images

Supplementary Figure 2e

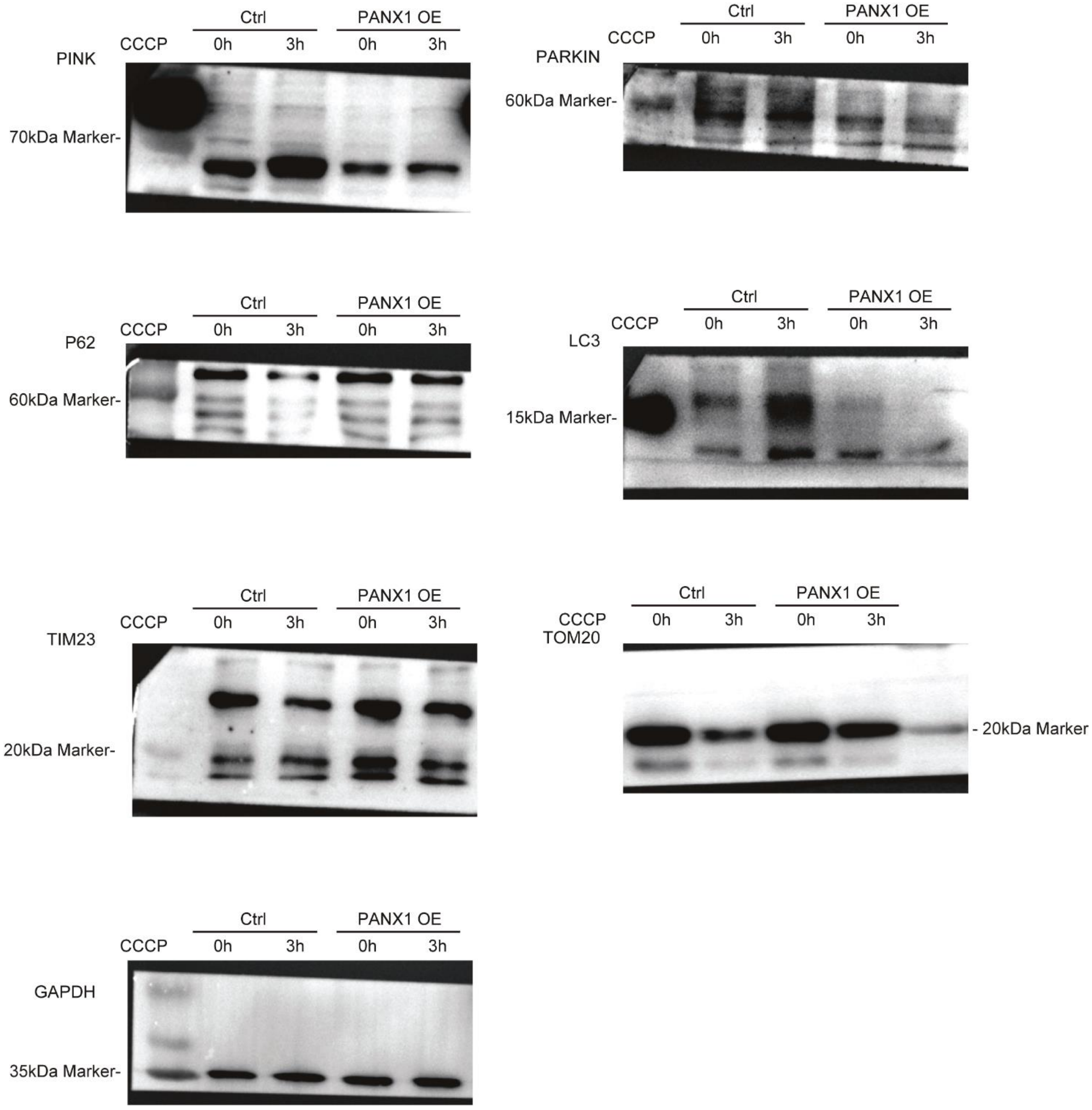

Supplement: Supplementary file 1 — Supplementary information [file 42003_2023_5226_MOESM1_ESM.pdf]
