# Supplementary material for: Pannexin 1 targets mitophagy to mediate renal ischemia/reperfusion injury
Source: Commun Biol. 2023 Aug 29;6:889. doi: 10.1038/s42003-023-05226-x (PMC10465551; doi:10.1038/s42003-023-05226-x)
Supplement: Supplementary file 2 — Description of Additional Supplementary Files [file 42003_2023_5226_MOESM2_ESM.pdf]

Description of Additional Supplementary Files

File name: Supplementary Data

Description: Numerical source data for Figs. 1, 2, 3, 4, 5, 6 and Supplementary Figs. 1, 2
