# Supplementary material for: Pannexin 1 targets mitophagy to mediate renal ischemia/reperfusion injury
Source: Commun Biol. 2023 Aug 29;6:889. doi: 10.1038/s42003-023-05226-x (PMC10465551; doi:10.1038/s42003-023-05226-x)
Supplement: Supplementary file 4 — Reporting Summary [file 42003_2023_5226_MOESM4_ESM.pdf]

Corresponding author(s): Zhiyong Peng
Last updated by author(s): Aug 4, 2023

Reporting Summary

Nature Portfolio wishes to improve the reproducibility of the work that we publish. This form provides structure for consistency and transparency in reporting. For further information on Nature Portfolio policies, see our Editorial Policies and the Editorial Policy Checklist.

Statistics

For all statistical analyses, confirm that the following items are present in the figure legend, table legend, main text, or Methods section.

n/a	Confirmed	
☐	☒	The exact sample size (*n*) for each experimental group/condition, given as a discrete number and unit of measurement
☐	☒	A statement on whether measurements were taken from distinct samples or whether the same sample was measured repeatedly
☐	☒	The statistical test(s) used AND whether they are one- or two-sided<br>*Only common tests should be described solely by name; describe more complex techniques in the Methods section.*
☐	☒	A description of all covariates tested
☐	☒	A description of any assumptions or corrections, such as tests of normality and adjustment for multiple comparisons
☐	☒	A full description of the statistical parameters including central tendency (e.g. means) or other basic estimates (e.g. regression coefficient) AND variation (e.g. standard deviation) or associated estimates of uncertainty (e.g. confidence intervals)
☐	☒	For null hypothesis testing, the test statistic (e.g. *F*, *t*, *r*) with confidence intervals, effect sizes, degrees of freedom and *P* value noted<br>*Give P values as exact values whenever suitable.*
☐	☒	For Bayesian analysis, information on the choice of priors and Markov chain Monte Carlo settings
☐	☒	For hierarchical and complex designs, identification of the appropriate level for tests and full reporting of outcomes
☐	☒	Estimates of effect sizes (e.g. Cohen's *d*, Pearson's *r*), indicating how they were calculated

Our web collection on statistics for biologists contains articles on many of the points above.

Software and code

Policy information about availability of computer code

Data collection	Statistical Package for the Social Sciences (version 22.0)
Data analysis	Statistical Package for the Social Sciences (version 22.0), and GraphPad Prism software (version 8.0)

For manuscripts utilizing custom algorithms or software that are central to the research but not yet described in published literature, software must be made available to editors and reviewers. We strongly encourage code deposition in a community repository (e.g. GitHub). See the Nature Portfolio guidelines for submitting code & software for further information.

Data

Policy information about availability of data

All manuscripts must include a data availability statement. This statement should provide the following information, where applicable:

- Accession codes, unique identifiers, or web links for publicly available datasets
- A description of any restrictions on data availability
- For clinical datasets or third party data, please ensure that the statement adheres to our policy

All data generated or analysis during this study are included in this published article [and its supplementary information files].

Research involving human participants, their data, or biological material

Policy information about studies with human participants or human data. See also policy information about sex, gender (identity/presentation), and sexual orientation and race, ethnicity and racism.

Reporting on sex and gender	Approved by the ethics committee of Central South Hospital of Wuhan University in accordance with the principles of the declaration of Helsinki, the patients who were transferred to the intensive care unit after cardiopulmonary bypass surgery in Zhongnan Hospital of Wuhan University from January 1 to June 30, 2019 were included in this study. Patients who had AKI, chronic kidney disease, end-stage renal disease, tumor and had received renal replacement therapy were excluded from the study. Patients aged under 18 years or over 80 years and those who did not fill in the consent form were also excluded. With the informed consent of the patient or their family members, we have confirmed the biological sex of the patient.
Reporting on race, ethnicity, or other socially relevant groupings	Approved by the ethics committee of Central South Hospital of Wuhan University in accordance with the principles of the declaration of Helsinki, the patients who were transferred to the intensive care unit after cardiopulmonary bypass surgery in Zhongnan Hospital of Wuhan University from January 1 to June 30, 2019 were included in this study. Patients who had AKI, chronic kidney disease, end-stage renal disease, tumor and had received renal replacement therapy were excluded from the study. Patients aged under 18 years or over 80 years and those who did not fill in the consent form were also excluded.
Population characteristics	See above
Recruitment	The patients who were transferred to the intensive care unit after cardiopulmonary bypass surgery in Zhongnan Hospital of Wuhan University from January 1 to June 30, 2019 were included in this study. Patients who had AKI, chronic kidney disease, end-stage renal disease, tumor and had received renal replacement therapy were excluded from the study. Patients aged under 18 years or over 80 years and those who did not fill in the consent form were also excluded.
Ethics oversight	Approved by the ethics committee of Central South Hospital of Wuhan University in accordance with the principles of the declaration of Helsinki.

Note that full information on the approval of the study protocol must also be provided in the manuscript.

Field-specific reporting

Please select the one below that is the best fit for your research. If you are not sure, read the appropriate sections before making your selection.

☒ Life sciences ☐ Behavioural & social sciences ☐ Ecological, evolutionary & environmental sciences

For a reference copy of the document with all sections, see nature.com/documents/nr-reporting-summary-flat.pdf

Life sciences study design

All studies must disclose on these points even when the disclosure is negative.

Sample size	This study was a prospective observational research study performed in a 58-bed closed ICU of a 3300-bed tertiary center. The patients who were transferred to the intensive care unit after cardiopulmonary bypass surgery in Zhongnan Hospital of Wuhan University from January 1 to June 30, 2019 were included in this study.
Data exclusions	Patients who had AKI, chronic kidney disease, end-stage renal disease, tumor and had received renal replacement therapy were excluded from the study. Patients aged under 18 years or over 80 years and those who did not fill in the consent form were also excluded.
Replication	This study was a prospective observational research study.
Randomization	This study was a prospective observational research study.
Blinding	This study was a prospective observational research study.

Reporting for specific materials, systems and methods

We require information from authors about some types of materials, experimental systems and methods used in many studies. Here, indicate whether each material, system or method listed is relevant to your study. If you are not sure if a list item applies to your research, read the appropriate section before selecting a response.

Materials & experimental systems

n/a	Involved in the study
☐	☒ Antibodies
☒	☐ Eukaryotic cell lines
☒	☐ Palaeontology and archaeology
☐	☒ Animals and other organisms
☒	☐ Clinical data
☒	☐ Dual use research of concern
☒	☐ Plants

Methods

n/a	Involved in the study
☒	☐ ChIP-seq
☐	☒ Flow cytometry
☒	☐ MRI-based neuroimaging

Antibodies

Antibodies used

PANX1 12595-1-AP Proteintech rabbit
PANX1 #91137 Cell Signaling Technology rabbit
PANX1 sc-293210 Santa Cruz mouse
GAPDH 60004-1-Ig Proteintech mouse
LC3B 18725-1-AP Proteintech rabbit
LC3 14600-1-AP Proteintech rabbit
P62 18420-1-AP Proteintech rabbit
TOM20 11802-1-AP Proteintech rabbit
TIM23 67535-1-Ig Proteintech mouse
PINK DF7742 Affinity rabbit
PARKIN AF0235 Affinity rabbit
Caspase 3 19677-1-AP Proteintech rabbit
Bax 50599-2-Ig Proteintech rabbit
Bcl2 26593-1-AP Proteintech rabbit
mTOR #2972 Cell Signaling Technology rabbit
Phospho-mTOR(Ser2448) #5536 Cell Signaling Technology rabbit
Phospho-mTOR(Ser2481) #2974 Cell Signaling Technology rabbit
Phospho-ULK1 AF4387 Affinity rabbit
Phospho-4E-BP1 AF3432 Affinity rabbit
p70 S6 Kinase AF6226 Affinity rabbit
ULK1 AF7588 Affinity rabbit
4E-BP1 AF4387 Affinity rabbit

Validation

The validation ofeach primary antibody for the species and application could be acquired in themanufacturer's website.

Animals and other research organisms

Policy information about studies involving animals; ARRIVE guidelines recommended for reporting animal research, and Sex and Gender in Research

Laboratory animals

C57BL/6J wild-type (WT) mice and PANX1 knockout (PANX1 -/-) mice were used in the study.

Wild animals

C57BL/6J wild-type (WT) mice and PANX1-/- mice were purchased from Gem Pharmatech Co. Ltd. (Nanjing, China), and were created from C57BL/6J wild-type mice and PANX1+/- mice.

Reporting on sex

All mice used for experiments were 8-10 weeks old males

Field-collected samples

All mice used for experiments were raised under specific pathogen-free and controlled temperature conditions with a 12h light/dark cycle.

Ethics oversight

All animal experiments were approved by The Animal Care and Use Committee of Zhongnan Hospital of Wuhan University. All animal experimental procedures were performed in accordance with national and EU guidelines. Additionally, all animals were provided humane care according to the ARRIVE guidelines.

Note that full information on the approval of the study protocol must also be provided in the manuscript.

Flow Cytometry

Plots

Confirm that:

☒ The axis labels state the marker and fluorochrome used (e.g. CD4-FITC).

☒ The axis scales are clearly visible. Include numbers along axes only for bottom left plot of group (a 'group' is an analysis of identical markers).

☒ All plots are contour plots with outliers or pseudocolor plots.

☒ A numerical value for number of cells or percentage (with statistics) is provided.

Methodology

Sample preparation	After trypsinization, 10^6 cells were collected, the cells were collected and analyzed by flow cytometry.
Instrument	cytoFlex S (BECKMAN)
Software	CytExpert
Cell population abundance	After trypsinization, 10^6 cells were collected, the cells were collected and analyzed by flow cytometry.
Gating strategy	Single parameter histograms were applied to density plots to exclude debris.<br>Unstained controls and single color stained controls, were used to defend boundaries between "positive" and "negative" staining cell populations

☒ Tick this box to confirm that a figure exemplifying the gating strategy is provided in the Supplementary Information.
